# Global, regional, and national mortality of larynx cancer from 1990 to 2021: results from the global burden of disease study

**DOI:** 10.1186/s12957-025-03720-6

**Published:** 2025-03-07

**Authors:** Deqian Han, Hoi Leong Lee, Qi Wei Oung, Chia Hau Lee

**Affiliations:** 1 Department of Oncology, West China School of Public Health and West China Fourth Hospital, Sichuan University, Chengdu, Sichuan 610041 China; 2 Faculty of Electronic Engineering & Technology, Universiti Malaysia Perlis, Arau, Perlis, 02600 Malaysia

**Keywords:** Larynx cancer, Mortality trends, Public health, Global disparities

## Abstract

**Background:**

Larynx cancer, a major upper respiratory tract malignancy, remains a global public health challenge, driven by smoking, alcohol use, and chronic inflammation, despite medical and public health advancements.

**Methods:**

Data from the Global Burden of Disease 2021 study were used to assess larynx cancer mortality trends from 1990 to 2021 across global, regional, and national levels. Death rates, absolute mortality numbers, and Estimated Annual Percentage Change (EAPC) were calculated.

**Results:**

Globally, the number of deaths from larynx cancer increased by 36.67% between 1990 and 2021, while death rates slightly declined, with an EAPC of -0.41. Males consistently accounted for the majority of deaths, with 100,393 deaths in 2021, though female mortality showed a larger percentage increase of 60.13% compared to 33.39% in males. Significant regional disparities were evident, with the highest death rates reported in Eastern Europe and Central Latin America, where countries like Bulgaria and Cuba recorded rates exceeding 6 per 100,000 population. In contrast, Oceania reported the lowest rates, below 0.5 per 100,000. The elderly (75 + years) experienced the largest increase in mortality, rising by 85.4%, while deaths among the 15–49 age group remained relatively stable. Additionally, larynx cancer death rates were correlated with SDI.

**Conclusion:**

Despite slight declines in global death rates, the absolute burden of larynx cancer has increased due to population growth and aging. Regional disparities emphasize the need for targeted interventions and improved healthcare access. This study offers valuable insights for policy and resource planning.

**Supplementary Information:**

The online version contains supplementary material available at 10.1186/s12957-025-03720-6.

## Introduction

Larynx cancer, as a common malignancy of the upper respiratory tract, has long posed a serious challenge to global public health [1, 2]. Its major risk factors include smoking, excessive alcohol consumption, chronic inflammation, and infection with human papillomavirus (HPV) [3]. The early symptoms of larynx cancer are often subtle, leading to many patients being diagnosed at advanced stages, which significantly increases the complexity of treatment and the mortality rate [4]. Despite ongoing advancements in medical technology and public health measures, such as early screening, treatment methods, and improved patient survival rates, the global burden of larynx cancer remains heavy, with significant differences in mortality rates across regions [5].

In recent years, increasing attention has been given to the role of HPV infection in the development of larynx cancer [6]. HPV, particularly high-risk types such as HPV16, has been identified as a key pathogenic factor, especially in younger populations and female patients, with its pathogenic role becoming more evident [7, 8]. Therefore, the occurrence of larynx cancer is no longer limited to traditional risk factors, and the impact of viral infections on its pathogenesis is increasingly significant [3, 9]. Despite these advancements, early diagnosis and treatment of larynx cancer continue to face numerous challenges [10]. Most developing countries lack sufficient medical resources, resulting in inadequate early screening for larynx cancer, and patients are often diagnosed only when symptoms become apparent [11, 12]. Even in some high-income countries, the treatment of larynx cancer still requires complex approaches such as radiation therapy, surgical resection, and chemotherapy, particularly for late-stage patients, where treatment outcomes are often limited [13, 14]. As a result, the mortality rate from larynx cancer remains high in many countries, especially in regions such as Asia, Eastern Europe, and Latin America [15, 16].

This study uses data from the Global Burden of Disease (GBD) 2021 to comprehensively analyze the trends in larynx cancer mortality from 1990 to 2021 at the global, regional, and national levels [17, 18]. Through detailed analysis of data from various regions and countries, this paper aims to identify the main factors influencing changes in larynx cancer mortality and provide valuable support for the formulation of more precise public health policies worldwide. In particular, targeted interventions and resource investment in high-burden areas will be key to reducing larynx cancer mortality.

## Methods

### Data source

This study utilized data from the GBD 2021 database, which provides comprehensive data on disease burden, including mortality, morbidity, and risk factors, across a wide range of regions and countries [19, 20]. The GBD study collects data from various sources such as national vital statistics, disease registries, and epidemiological studies [19, 20]. For this analysis, data on larynx cancer mortality were extracted from the GBD 2021 dataset, spanning from 1990 to 2021. Mortality data were stratified by region, country, sex, and age group, with specific age groups of 15–49 years, 50–74 years, and 75 + years analyzed. This allows for a detailed analysis of trends in larynx cancer mortality across different demographics. Due to the lack of available data for the 0–14 age group, and considering that larynx cancer is rare in childhood and typically associated with different etiological factors than those in adults (such as smoking and alcohol consumption), this analysis focused on the adult population [21]. The data can be accessed through the GBD results tool at http://ghdx.healthdata.org/gbd-results-tool.

### Disease definition

Larynx cancer is defined by the International Classification of Diseases (ICD) codes C32-C32.9 in the ICD-10, which refer to malignant neoplasms of the larynx, including all forms of cancer affecting this region. The corresponding ICD-9 codes are 161-161.9 and V10.21, the latter indicating a history of laryngeal cancer [22]. These codes are widely used in hospital and claims analyses to identify and track cases of larynx cancer in clinical records, making it possible to monitor trends in diagnosis, treatment, and outcomes. Larynx cancer falls under the broader category of upper respiratory tract malignancies and is primarily associated with risk factors such as smoking, excessive alcohol consumption, and HPV infection [23]. The standardized use of ICD codes ensures consistency in identifying larynx cancer cases across healthcare settings, which is essential for epidemiological studies and health policy formulation [24].

### Statistical analysis

All estimates of larynx cancer deaths and cases were accompanied by 95% uncertainty intervals (UIs) based on the GBD framework. These UIs were derived from 1,000 iterations of data sampling, with the lower and upper bounds corresponding to the 25th and 975th ranked values, respectively. To assess trends in larynx cancer deaths from 1990 to 2021, the Estimated Annual Percentage Change (EAPC) was calculated. EAPC was derived by fitting a regression line to the natural logarithm of the death rates over time, using the formula: y = α + βx + ε, where y represents the natural logarithm of the death rate (ln(rate)), and x is the calendar year. The EAPC itself was then computed as: EAPC = 100 × (exp(β) − 1), where β is the slope coefficient from the regression, and exp(β) is the exponentiated value of β. The 95% confidence interval (CI) for the EAPC was derived from the regression model. An increase in death rate was indicated if both the EAPC estimate and its lower 95% CI were greater than zero. Conversely, a decrease was indicated if both the upper bound of the EAPC estimate and its 95% CI were less than zero. If neither condition was met, the rate was considered stable.

Additionally, correlation analysis was performed to examine the relationship between larynx cancer death and case rates and the Socio-Demographic Index (SDI) at global, regional, and country levels. The Pearson correlation coefficient (ρ) was used to assess the strength and direction of these relationships, with statistical significance set at *p* < 0.05. All analyses were conducted and visualized using R software.

## Results

### Global trends in larynx cancer deaths

**Fig. 1:**
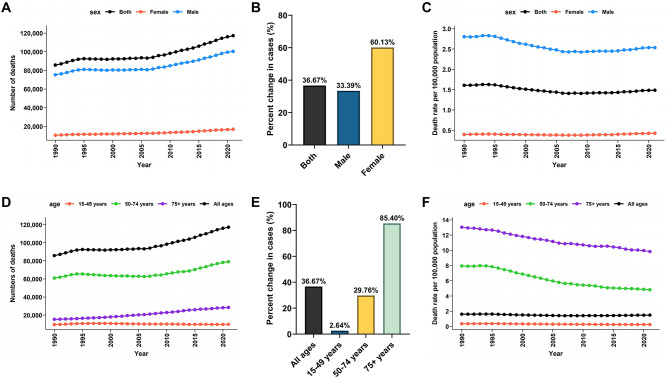
Global burden of larynx cancer deaths from 1990 to 2021 by sex and age groups. (**A**) Number of cases by sex (both male and female) from 1990 to 2021. (**B**) Percentage change in the number of cases by sex from 1990 to 2021. (**C**) Death rates by sex (both male and female) from 1990 to 2021. (**D**) Number of cases by age group (10–14, 15–19, 20–24 years) from 1990 to 2021. (**E**) Percentage change in the number of cases by age group from 1990 to 2021. (**F**) Death rates by age group (10–14, 15–19, 20–24 years) from 1990 to 2021

Globally, the number of larynx cancer deaths increased significantly from 1990 to 2021, rising by 36.67% (95% UI: 25–49%), from 85,790 deaths (95% UI: 80,409 to 91,208) in 1990 to 117,252 deaths (95% UI: 109,355 to 125,952) in 2021 (Fig. 1A-C; Table 1). However, the global death rate decreased slightly over the same period, from 1.61 per 100,000 population (95% UI: 1.51 to 1.71) in 1990 to 1.49 per 100,000 population (95% UI: 1.39 to 1.6) in 2021. The EAPC for the global death rate was − 0.41 (95% CI: -0.53 to -0.29) (Fig. 1D-F; Table 1), reflecting a small but statistically significant decline over time.

**Table 1:** Global and regional death rates and trends of larynx cancer from 1990 to 2021

Characteristics	Cases_1990	Rates_1990	Cases_2021	Rates_2021	Cases_change %	EAPC_CI
**Global**	85,790(80409 to 91208)	1.61(1.51 to 1.71)	117,252(109355 to 125952)	1.49(1.39 to 1.6)	36.67(25 to 49)	-0.41(-0.53 to -0.29)
**Sex**						
Male	75,262(70749 to 80447)	2.8(2.63 to 3)	100,393(93351 to 108830)	2.54(2.36 to 2.75)	33.39(21 to 46)	-0.47(-0.6 to -0.35)
Female	10,528(8183 to 11773)	0.4(0.31 to 0.44)	16,859(14209 to 19876)	0.43(0.36 to 0.51)	60.13(40 to 99)	0.1(-0.02 to 0.22)
**Age**						
15–49 years	9523(8683 to 10212)	0.35(0.32 to 0.38)	9775(8898 to 10869)	0.25(0.23 to 0.28)	2.64(-9 to 15)	-1.47(-1.58 to -1.36)
50–74 years	60,962(57415 to 64871)	7.95(7.49 to 8.46)	79,102(73871 to 85234)	4.82(4.5 to 5.19)	29.76(18 to 42)	-1.9(-2.02 to -1.78)
75 + years	15,304(14143 to 16318)	13.04(12.05 to 13.91)	28,375(25586 to 30537)	9.83(8.87 to 10.58)	85.4(72 to 100)	-0.93(-0.98 to -0.89)
**SDI regions**						
High SDI	17,522(16916 to 18077)	1.99(1.92 to 2.06)	15,698(14580 to 16475)	1.43(1.33 to 1.51)	-10.41(-15 to -6)	-1.22(-1.33 to -1.1)
High-middle SDI	27,382(26032 to 28742)	2.57(2.45 to 2.7)	25,890(23623 to 28384)	1.99(1.81 to 2.18)	-5.45(-15 to 4)	-1.14(-1.27 to -1.02)
Middle SDI	19,864(18170 to 21395)	1.15(1.05 to 1.24)	35,944(32307 to 39867)	1.47(1.32 to 1.63)	80.95(59 to 104)	0.69(0.59 to 0.79)
Low-middle SDI	15,754(13442 to 18423)	1.36(1.16 to 1.59)	30,466(27265 to 34217)	1.59(1.42 to 1.78)	93.39(61 to 131)	0.49(0.38 to 0.61)
Low SDI	5133(4033 to 6314)	1.02(0.8 to 1.26)	9099(7802 to 10534)	0.81(0.7 to 0.94)	77.28(48 to 116)	-0.88(-1.03 to -0.74)
**Location**						
Andean Latin America	237(207 to 272)	0.62(0.54 to 0.72)	367(286 to 458)	0.56(0.43 to 0.69)	55.05(17 to 101)	-0.39(-0.62 to -0.16)
Australasia	277(255 to 305)	1.37(1.26 to 1.51)	266(233 to 297)	0.86(0.75 to 0.96)	-4.01(-15 to 10)	-1.59(-1.69 to -1.49)
Caribbean	748(690 to 814)	2.12(1.95 to 2.31)	1457(1264 to 1700)	3.07(2.66 to 3.58)	94.8(66 to 131)	1.37(1.28 to 1.47)
Central Asia	1378(1321 to 1442)	1.99(1.91 to 2.08)	999(891 to 1118)	1.04(0.93 to 1.17)	-27.47(-36 to -18)	-2.22(-2.36 to -2.08)
Central Europe	5604(5326 to 5920)	4.48(4.26 to 4.73)	5322(4877 to 5764)	4.62(4.23 to 5)	-5.03(-14 to 5)	0.01(-0.08 to 0.09)
Central Latin America	1645(1587 to 1699)	1(0.97 to 1.03)	2310(2030 to 2634)	0.91(0.8 to 1.04)	40.45(24 to 59)	-0.69(-0.8 to -0.58)
Central Sub-Saharan Africa	370(264 to 493)	0.67(0.48 to 0.9)	758(559 to 982)	0.55(0.41 to 0.72)	104.87(47 to 187)	-0.7(-0.93 to -0.46)
East Asia	13,217(10939 to 15503)	1.09(0.9 to 1.27)	20,328(16104 to 25532)	1.38(1.09 to 1.73)	53.8(15 to 101)	0.78(0.63 to 0.93)
Eastern Europe	9604(9281 to 9919)	4.24(4.1 to 4.38)	6043(5361 to 6778)	2.92(2.59 to 3.28)	-37.08(-44 to -29)	-1.92(-2.15 to -1.69)
Eastern Sub-Saharan Africa	1318(1029 to 1608)	0.69(0.54 to 0.84)	2200(1725 to 2831)	0.52(0.4 to 0.66)	66.99(36 to 114)	-1.16(-1.32 to -1)
High-income Asia Pacific	1665(1460 to 1837)	0.96(0.84 to 1.06)	1634(1403 to 1815)	0.88(0.76 to 0.98)	-1.85(-16 to 15)	-0.75(-0.91 to -0.59)
High-income North America	4685(4503 to 4812)	1.66(1.6 to 1.71)	5057(4755 to 5287)	1.37(1.28 to 1.43)	7.94(4 to 12)	-0.87(-0.97 to -0.77)
North Africa and Middle East	3935(3271 to 4564)	1.16(0.96 to 1.35)	6992(6119 to 7962)	1.12(0.98 to 1.28)	77.68(45 to 114)	-0.18(-0.3 to -0.07)
Oceania	15(11 to 20)	0.23(0.17 to 0.31)	34(26 to 45)	0.24(0.19 to 0.32)	121.53(70 to 200)	0.06(0 to 0.13)
South Asia	19,009(16065 to 22229)	1.74(1.47 to 2.03)	37,433(32670 to 42841)	2.03(1.77 to 2.32)	96.93(57 to 142)	0.37(0.21 to 0.53)
Southeast Asia	3262(2831 to 3682)	0.7(0.61 to 0.79)	7055(6140 to 8307)	1.01(0.88 to 1.19)	116.24(84 to 158)	1.12(1.04 to 1.2)
Southern Latin America	1429(1331 to 1550)	2.88(2.69 to 3.13)	1176(1071 to 1282)	1.74(1.58 to 1.89)	-17.7(-27 to -7)	-1.62(-1.72 to -1.52)
Southern Sub-Saharan Africa	538(451 to 697)	1.03(0.86 to 1.33)	1022(899 to 1165)	1.27(1.12 to 1.45)	90.04(55 to 128)	0.48(0.23 to 0.74)
Tropical Latin America	2599(2492 to 2705)	1.7(1.63 to 1.77)	5593(5230 to 5917)	2.46(2.3 to 2.6)	115.18(103 to 128)	1.27(1.18 to 1.36)
Western Europe	13,231(12685 to 13722)	3.44(3.3 to 3.57)	9085(8334 to 9651)	2.08(1.91 to 2.21)	-31.34(-36 to -27)	-1.67(-1.82 to -1.51)
Western Sub-Saharan Africa	1024(814 to 1269)	0.53(0.42 to 0.66)	2119(1707 to 2568)	0.43(0.35 to 0.52)	107.04(59 to 179)	-0.6(-0.64 to -0.56)

Data in parentheses are 95% uncertainty intervals for cases and rates, and 95% confidence intervals for EAPC. Rate is expressed as per 100,000 population

### Age- and sex-specific mortality trends

Males accounted for the majority of larynx cancer deaths throughout the study period. In 2021, there were 100,393 male deaths (95% UI: 93,351 to 108,830) compared to 16,859 female deaths (95% UI: 14,209 to 19,876). Despite the higher absolute number of deaths in males, the percentage increase in deaths was more pronounced in females, rising by 60.13% (95% UI: 40–99%), compared to 33.39% (95% UI: 21–46%) in males (Fig. 1A-B; Table 1). The death rate for males decreased from 2.8 per 100,000 population (95% UI: 2.63 to 3) in 1990 to 2.54 per 100,000 population (95% UI: 2.36 to 2.75) in 2021, with an EAPC of -0.47 (95% CI: -0.6 to -0.35). In contrast, the death rate for females slightly increased, rising from 0.4 per 100,000 population (95% UI: 0.31 to 0.44) in 1990 to 0.43 per 100,000 population (95% UI: 0.36 to 0.51) in 2021, with an EAPC of 0.1 (95% CI: -0.02 to 0.22) (Fig. 1C; Table 1).

Larynx cancer deaths also varied significantly across age groups. The largest increase was observed in the 75 + years age group, where deaths rose by 85.4% (95% UI: 72–100%), from 15,304 deaths (95% UI: 14,143 to 16,318) in 1990 to 28,375 deaths (95% UI: 25,586 to 30,537) in 2021. Deaths in the 50–74 years group also increased, rising by 29.76% (95% UI: 18–42%) (Fig. 1D-E; Table 1). In contrast, deaths in the 15–49 years age group remained relatively stable, with an increase of only 2.64% (95% UI: -9–15%), from 9,523 deaths (95% UI: 8,683 to 10,212) in 1990 to 9,775 deaths (95% UI: 8,898 to 10,869) in 2021 (Fig. 1E; Table 1). The death rate in this group decreased from 0.35 per 100,000 population (95% UI: 0.32 to 0.38) in 1990 to 0.25 per 100,000 population (95% UI: 0.23 to 0.28) in 2021, remaining the lowest across all age groups (Fig. 1F; Table 1).

### Regional variation in larynx cancer mortality rates

**Fig. 2:**
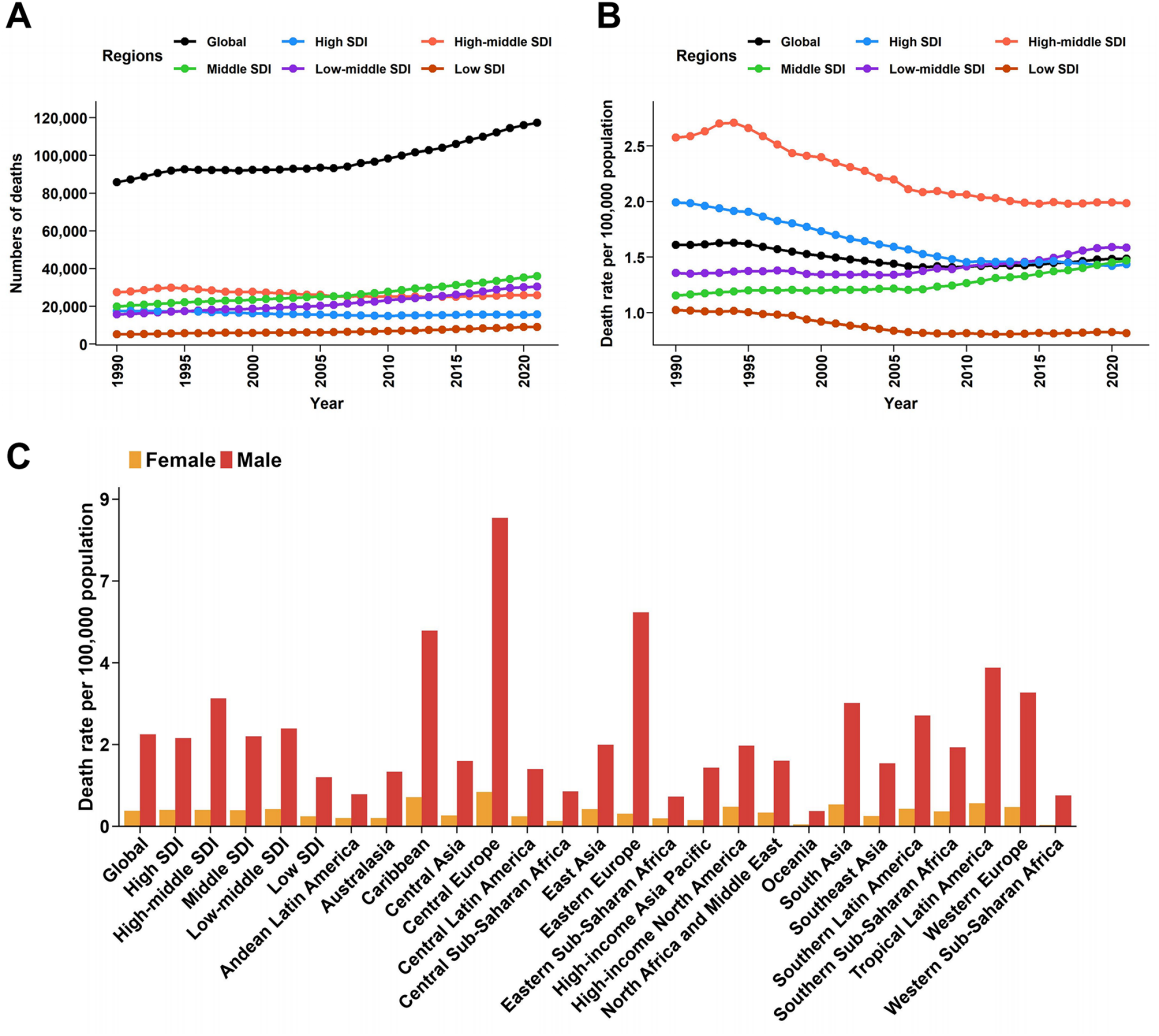
Regional trends in larynx cancer deaths. (**A**) New cases of larynx cancer in different SDI regions from 1990 to 2021. (**B**) Death rates of larynx cancer in different SDI regions from 1990 to 2021. (**C**) Death rates of larynx cancer by sex in 2021, globally, in SDI regions, and in geographical regions

Larynx cancer mortality rates varied significantly across regions and SDI levels from 1990 to 2021 (Fig. 2A-B; Table 1). In high SDI regions, such as Western Europe and High-income Asia Pacific, the death rates declined significantly over the study period. For instance, in high SDI regions, the death rate decreased from 1.99 per 100,000 population (95% UI: 1.92 to 2.06) in 1990 to 1.43 per 100,000 population (95% UI: 1.33 to 1.51) in 2021, with an EAPC of -1.22 (95% CI: -1.33 to -1.1) (Fig. 2A-B; Table 1). In contrast, low and low-middle SDI regions saw an increase in the number of deaths, despite slight declines in death rates. For example, in low SDI regions, the death rate decreased from 1.02 per 100,000 population (95% UI: 0.8 to 1.26) in 1990 to 0.81 per 100,000 population (95% UI: 0.7 to 0.94) in 2021, but the number of deaths rose by 77.28% (95% UI: 48–116%), reflecting population growth and limited healthcare access (Fig. 2A-B; Table 1). Middle SDI regions, such as South Asia and Southeast Asia, experienced increases in both death rates and the number of deaths. The death rate in middle SDI regions increased from 1.15 per 100,000 population (95% UI: 1.05 to 1.24) in 1990 to 1.47 per 100,000 population (95% UI: 1.32 to 1.63) in 2021, with an EAPC of 0.69 (95% CI: 0.59 to 0.79) (Fig. 2A-B; Table 1).

At the regional level, the highest death rates in 2021 were observed in the Caribbean, Central Europe, and Eastern Europe, with rates of 3.07 per 100,000 population (95% UI: 2.66 to 3.58), 4.62 per 100,000 population (95% UI: 4.23 to 5), and 2.92 per 100,000 population (95% UI: 2.59 to 3.28), respectively. Despite a declining trend in death rates, both Central Europe and Eastern Europe showed significant reductions in deaths, with EAPCs of 0.01 (95% CI: -0.08 to 0.09) and − 1.92 (95% CI: -2.15 to -1.69), respectively (Fig. 2C; Table 1). In contrast, the lowest death rates in 2021 were recorded in Eastern Sub-Saharan Africa, Oceania, and Western Sub-Saharan Africa, with rates of 0.52 per 100,000 population (95% UI: 0.4 to 0.66), 0.24 per 100,000 population (95% UI: 0.19 to 0.32), and 0.43 per 100,000 population (95% UI: 0.35 to 0.52), respectively (Fig. 2C; Table 1). These regions experienced either stable or modest declines in death rates over the study period, reflecting challenges in healthcare access and prevention efforts.

### Country-level differences in larynx cancer deaths

**Fig. 3:**
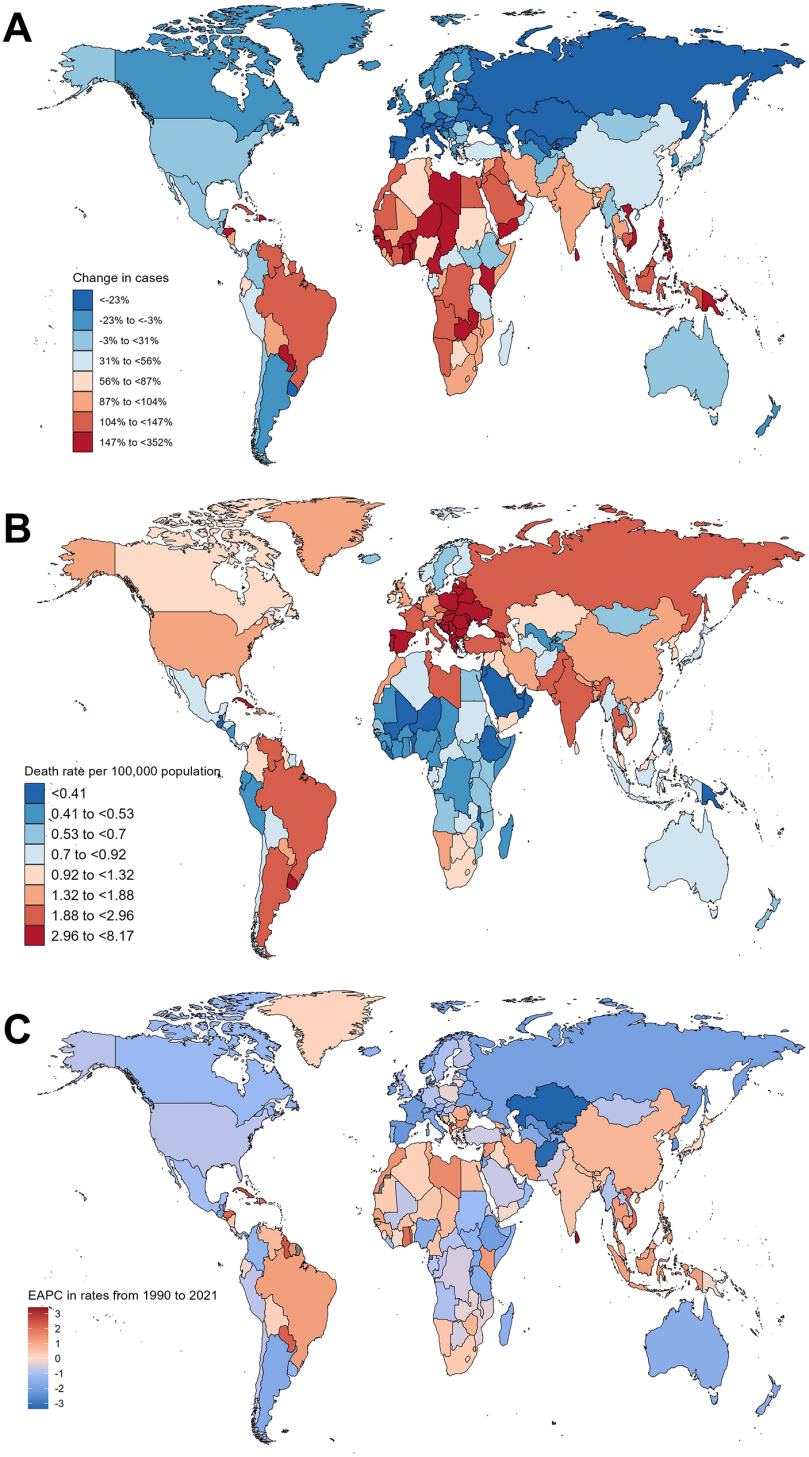
Global distribution of larynx cancer deaths in 204 countries. (**A**) Larynx cancer death rates by country in 2021. (**B**) Percentage change in larynx cancer deaths across countries from 1990 to 2021. (C) EAPC of larynx cancer deaths from 1990 to 2021

Significant country-level differences in larynx cancer deaths were observed between 1990 and 2021. India, China, and the United States reported the highest number of deaths in 2021, with cases reaching 28,330 (95% UI: 24,664 to 32,829), 19,799 (95% UI: 15,580 to 25,023), and 4,620 (95% UI: 4,340 to 4,836), respectively. Other countries with notably high case numbers included Brazil, with 5,497 deaths (95% UI: 5,144 to 5,812), and Pakistan, with 5,617 deaths (95% UI: 4,106 to 7,453) (Supplementary Table S1). These countries collectively accounted for a substantial proportion of global larynx cancer deaths due to their large populations. In contrast, several smaller nations, such as Niue, Cook Islands, and American Samoa, reported fewer than 10 deaths annually, reflecting their small population sizes and lower overall burden of disease (Supplementary Table S1). Interestingly, some countries with a smaller population demonstrated rapid increases in death rates. For example, Timor-Leste and Togo saw dramatic increases in deaths by 183.7% (95% UI: 95.71–320.65%) and 289.8% (95% UI: 156.41–473.24%), respectively, highlighting the rising burden of larynx cancer in low-resource settings (Fig. 3A and Supplementary Table S1). This underscores the importance of targeted prevention and control strategies in these regions.

Despite the high absolute number of deaths, trends in death rates varied across countries. For example, countries such as India, China, and Vietnam experienced a significant increase in deaths, with India showing a 100.9% rise (95% UI: 57.89–149.21%) and an EAPC of 0.50 (Fig. 3A-C and Supplementary Table S1). Similarly, Vietnam reported a 163.4% increase in deaths (95% UI: 81.17–279.16%), with an EAPC of 2.00. In contrast, countries like Kazakhstan, Belgium, and Russia experienced notable reductions in larynx cancer deaths, with EAPCs of -3.37, -2.45, and − 2.02, respectively (Fig. 3A-C and Supplementary Table S1). The steepest decline was observed in Kazakhstan, where deaths dropped by 48.1% (95% UI: -55.73% to -38.90%) from 1990 to 2021 (Fig. 3A and Supplementary Table S1).

Countries with the highest death rates in 2021 were predominantly located in Eastern Europe and Central Latin America. Bulgaria (6.20 per 100,000 population), Cuba (8.17 per 100,000 population), Monaco (7.90 per 100,000 population), Montenegro (7.42 per 100,000 population), and Romania (5.54 per 100,000 population) recorded the highest rates (Fig. 3B and Supplementary Table S1). These elevated rates reflect the persistence of major risk factors, such as tobacco and alcohol consumption, in these regions. In contrast, countries in Oceania, such as Fiji, Vanuatu, and Kiribati, had the lowest death rates, all below 0.5 per 100,000 population (Fig. 3B and Supplementary Table S1), reflecting potentially different epidemiological patterns and risk factor exposure.

### Correlation with SDI

**Fig. 4:**
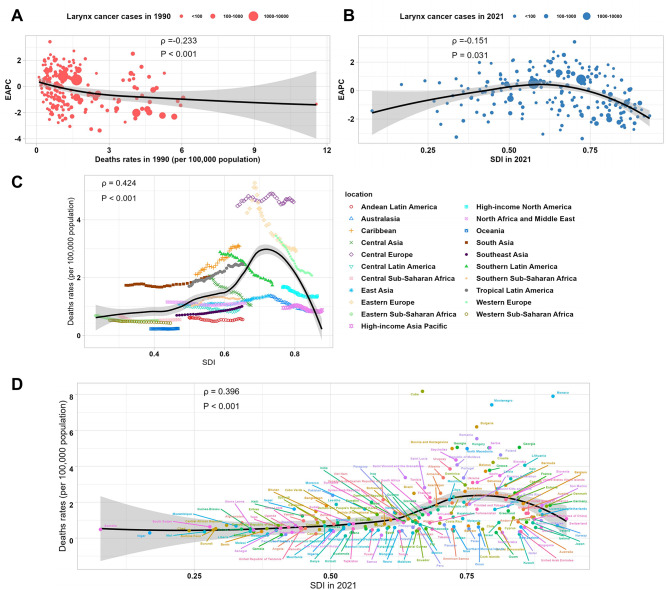
Correlation between larynx cancer deaths, EAPC, and SDI. (**A**) Correlation between larynx cancer death rates and EAPC in 1990. (**B**) Correlation between the SDI and larynx cancer death rates in 2021. (**C**) Relationship between between larynx cancer death rates and SDI at the regional level. (**D**) Relationship between between larynx cancer death rates and SDI at the country level

The correlation analysis highlighted significant relationships between larynx cancer deaths and the SDI across various levels. At the global level, a moderate positive correlation was observed between EAPC and death rates in 1990 (ρ = 0.233, *P* < 0.001), indicating that regions with higher initial death rates experienced slower declines in mortality trends over time (Fig. 4A). A weaker but significant positive correlation was also found between EAPC and SDI in 2021 (ρ = 0.151, *P* = 0.031), suggesting that socio-economic development plays a role in shaping the temporal trends of larynx cancer mortality (Fig. 4B). At the regional level, a strong positive correlation (ρ = 0.424, *P* < 0.001) was identified between death rates and SDI in 2021, with regions such as Central Latin America and Eastern Europe, characterized by moderate SDI values, exhibiting the highest death rates. This pattern likely reflects the persistence of major risk factors, such as tobacco and alcohol consumption, in these socio-economic settings (Fig. 4C). At the national level, a similar trend was evident, with a moderate positive correlation (ρ = 0.396, *P* < 0.001) between death rates and SDI in 2021. Countries with medium-to-high SDI values generally reported higher death rates compared to those with very low or very high SDI values, reflecting differences in lifestyle risk factors, healthcare access, and disease detection rates (Fig. 4D). These findings underscore the complex interplay between socio-economic development and larynx cancer mortality patterns.

## Discussion

Larynx cancer continues to be a major global public health concern, with significant disparities in mortality trends across regions and countries [25, 26]. This study provides a detailed analysis of larynx cancer mortality from 1990 to 2021 using data from the GBD 2021, revealing critical insights into the patterns and determinants of this disease. Despite advancements in medical technologies, public health interventions, and increased awareness of modifiable risk factors, such as smoking and alcohol consumption, the global burden of larynx cancer remains significant [27, 28]. While the overall global death rate has slightly declined over the last three decades, the absolute number of deaths has increased substantially due to population growth and aging, highlighting the persistent challenges in controlling this malignancy.

The uneven trends in larynx cancer mortality reflect regional and country-level disparities driven by socio-economic, cultural, and healthcare factors. High-income countries have demonstrated substantial progress in reducing mortality, largely attributable to effective public health campaigns, improved access to healthcare, and advancements in early detection and treatment technologies [29]. For instance, countries in Western Europe and High-income Asia Pacific experienced significant reductions in death rates, supported by declining smoking prevalence and improvements in cancer care infrastructure [30]. However, despite the quality-of-life benefits of chemoradiation, studies have raised concerns about its long-term oncological efficacy, with mixed results regarding survival rates compared to traditional surgical methods like total laryngectomy [31, 32]. Regions such as Eastern Europe, Central Latin America, and parts of Asia continue to report high mortality rates. Notably, countries like Bulgaria, Cuba, and Romania had the highest death rates in 2021, reflecting the sustained impact of risk factors like smoking and alcohol consumption, compounded by limited access to advanced treatments like chemoradiation and early detection technologies [32]. Conversely, Oceania exhibited some of the lowest death rates, suggesting variations in risk factor exposure and disease patterns.

At the national level, populous countries like India and China contributed significantly to the global burden of laryngeal cancer, with over 48,000 deaths collectively in 2021. However, trends in laryngeal cancer mortality differ from those of other head and neck cancers, such as oral cavity and pharyngeal cancers [33, 34]. While the incidence of HPV-related oropharyngeal cancers has risen in high-income regions like the US and Western Europe, contributing to a decline in mortality, laryngeal cancer remains primarily driven by smoking and alcohol consumption, showing only modest improvements in death rates [35]. Moreover, early detection and advancements in treatment for oral cavity and pharyngeal cancers have resulted in more significant declines in mortality compared to laryngeal cancer [36]. In contrast, smaller nations like Timor-Leste and Togo, despite their limited populations, have seen rapid increases in mortality from both laryngeal and other head and neck cancers due to high smoking rates and limited access to healthcare. These trends highlight the need for tailored public health interventions that address both traditional risk factors, such as smoking, and emerging factors like HPV in different regions.

Emerging research highlights the role of HPV infection in larynx cancer pathogenesis, particularly in younger populations and female patients [37, 38]. High-risk HPV subtypes, such as HPV16, are increasingly recognized as key etiological factors, shifting the understanding of larynx cancer from traditional risk factors alone to a more complex interplay of viral and environmental factors [37, 39]. This shift underscores the importance of integrating HPV vaccination into public health strategies aimed at reducing the incidence and mortality of larynx cancer [39, 40]. Furthermore, advancements in genomic and molecular profiling have provided deeper insights into the biological mechanisms underlying larynx cancer, paving the way for the development of targeted therapies and precision medicine approaches [41, 42]. It is worth noting that in regions with high laryngeal cancer mortality, such as Brazil, smoking remains the primary risk factor, and the contribution of HPV to laryngeal cancer is limited. In contrast, HPV-related oropharyngeal cancers, typically occurring in younger individuals, tend to have a better prognosis [43]. This regional disparity underscores the need for targeted prevention and treatment strategies based on the dominant risk factors in different populations.

While our analysis primarily focused on mortality rates, it is important to note that other epidemiological indicators, such as incidence and prevalence, could provide a more comprehensive evaluation of the disease burden. Regions with high mortality rates may also show significant differences in incidence and prevalence, which could reflect disparities in healthcare access, early detection, and risk factor exposure. Utilizing a broader range of epidemiological metrics, including incidence, prevalence, and disability-adjusted life years (DALYs), could offer deeper insights into the global disparities in larynx cancer outcomes and better inform strategies to improve healthcare access and treatment in high-burden regions.

Age and sex remain critical determinants of larynx cancer mortality [44]. While males consistently account for the majority of deaths, the percentage increase in female mortality suggests a shift in risk factor exposure, possibly due to rising smoking prevalence among women in certain regions [27, 29]. The disproportionate increase in deaths among older adults (75 + years) highlights the impact of population aging and cumulative exposure to carcinogens, whereas the stable trends in younger age groups reflect the comparatively lower burden in these populations [45, 46]. These findings call for age-specific and sex-specific public health interventions to address the unique needs of different demographic groups. Although this study is limited by the reliance on secondary data from the GBD study and may not fully capture regional variations or recent healthcare trends, with similar limitations as other GBD studies [19, 26, 47, 48], its strengths lie in its comprehensive analysis of long-term trends across multiple regions and demographics, offering valuable insights for targeted public health strategies worldwide.

## Conclusions

In conclusion, larynx cancer remains a significant global health challenge with marked disparities across regions and countries. Although global death rates have shown a slight decline, the absolute number of deaths continues to increase, particularly in regions like Eastern Europe and Central Latin America, due to persistent risk factors such as smoking and alcohol consumption. Addressing these disparities requires targeted public health interventions, improved healthcare access, and strengthened prevention and control strategies to reduce the burden of larynx cancer worldwide.

## Electronic supplementary material

Below is the link to the electronic supplementary material.

Supplementary Material 1

## Data Availability

The data are available from the Global Burden of Disease Results Tool of the Global Health Data Exchange (http://ghdx.healthdata.org/).

## Abbreviations

DALYs: Disability-adjusted life years
GBD: Global burden of disease
UI: Uncertainty interval
EAPC: Estimated annual percentage change
CI: Confidence interval
SDI: Socio-demographic index
ICD: International classification of diseases

## References

[1] Divakar P, Davies L. Trends in incidence and mortality of Larynx Cancer in the US. JAMA Otolaryngol Head Neck Surg. 2023;149:34–41. 36394832 10.1001/jamaoto.2022.3636 PMC9673027

[2] Gumbarewicz E, Tylżanowski P, Łuszczki J, Kałafut J, Czerwonka A, Szumiło J, et al. Differential molecular response of larynx cancer cell lines to combined VPA/CDDP treatment. Am J Cancer Res. 2021;11:2821–37. 34249430 PMC8263637

[3] Liberale C, Soloperto D, Marchioni A, Monzani D, Sacchetto L. Updates on Larynx Cancer: risk factors and oncogenesis. Int J Mol Sci. 2023;24:12913. 37629093 10.3390/ijms241612913 PMC10454133

[4] Kim TE, Chang J-E. Recent studies in photodynamic therapy for Cancer Treatment: from Basic Research to clinical trials. Pharmaceutics. 2023;15:2257. 37765226 10.3390/pharmaceutics15092257 PMC10535460

[5] Arboleda LPA, de Carvalho GB, Santos-Silva AR, Fernandes GA, Vartanian JG, Conway DI, et al. Squamous cell carcinoma of the oral cavity, Oropharynx, and larynx: a scoping review of Treatment guidelines Worldwide. Cancers (Basel). 2023;15:4405. 37686681 10.3390/cancers15174405 PMC10486835

[6] Panuganti BA, Finegersh A, Flagg M, Tu X, Orosco R, Weissbrod PA, et al. Prognostic significance of HPV Status in laryngeal squamous cell carcinoma: a large-Population Database Study. Otolaryngol Head Neck Surg. 2021;165:113–21. 33256521 10.1177/0194599820976178

[7] Davidson SM, Ko HC, Harari PM, Wieland AM, Chen S, Baschnagel AM, et al. Impact of HPV Status on the prognostic potential of the AJCC staging system for Larynx Cancer. Otolaryngol Head Neck Surg. 2018;159:456–65. 29611770 10.1177/0194599818766035 PMC7141595

[8] Delagranda A, Leterme G, Chirpaz E, Ferdynus C, Fernandez C, Rubin F. Epidemiological features of cancers of the oral cavity, oropharynx, hypopharynx and larynx cancer in Réunion Island. Eur Ann Otorhinolaryngol Head Neck Dis. 2018;135:175–81. 29673737 10.1016/j.anorl.2018.01.008

[9] Scheel A, Bellile E, McHugh JB, Walline HM, Prince ME, Urba S, et al. Classification of TP53 mutations and HPV predict survival in advanced larynx cancer. Laryngoscope. 2016;126:E292–299. 27345657 10.1002/lary.25915 PMC5002993

[10] Kim HJ, Boyd J, Dunphy F, Lowe V. F-18 FDG PET scan after radiotherapy for early-stage larynx cancer. Clin Nucl Med. 1998;23:750–2. 9814562 10.1097/00003072-199811000-00006

[11] de Jong MC, Pramana J, van der Wal JE, Lacko M, Peutz-Kootstra CJ, de Jong JM, et al. CD44 expression predicts local recurrence after radiotherapy in larynx cancer. Clin Cancer Res. 2010;16:5329–38. 20837694 10.1158/1078-0432.CCR-10-0799

[12] Sher DJ, Timmerman RD, Nedzi L, Ding C, Pham N-L, Zhao B, et al. Phase 1 fractional dose-escalation study of Equipotent Stereotactic Radiation Therapy regimens for Early-Stage Glottic Larynx Cancer. Int J Radiat Oncol Biol Phys. 2019;105:110–8. 30880269 10.1016/j.ijrobp.2019.03.010

[13] Tollosa DN, Zendehdel K, Procopio A, Cederström A, Boffetta P, Pukkala E, et al. Cancer mortality by country of birth and cancer type in Sweden: a 25-year registry-based cohort study. Cancer Med. 2024;13:e70020. 39016445 10.1002/cam4.70020 PMC11253184

[14] Nikkilä R, Haapaniemi A, Carpén T, Pukkala E, Mäkitie A. Laryngeal cancer relative survival trends from 1972 to 2021 in the nordic countries. Acta Oncol. 2024;63:612–9. 39099322 10.2340/1651-226X.2024.40823 PMC11332480

[15] Joko-Fru WY, Bardot A, Bukirwa P, Amidou S, N’da G, Woldetsadik E, et al. Cancer survival in sub-saharan Africa (SURVCAN-3): a population-based study. Lancet Glob Health. 2024;12:e947–59. 38762297 10.1016/S2214-109X(24)00130-X PMC11126368

[16] Zhong R, Cai X, Li J, Chen P, Wang R, Li X, et al. Asian, regional, and national burdens of respiratory tract cancers and associated risk factors from 1990 to 2019: a systematic analysis for the global burden of disease study 2019. Chin Med J Pulm Crit Care Med. 2023;1:249–58. 39171284 10.1016/j.pccm.2023.11.002 PMC11332864

[17] Ferguson LD, Molenberghs G, Verbeke G, Rahimi K, Rao S, McInnes IB, et al. Gout and incidence of 12 cardiovascular diseases: a case-control study including 152 663 individuals with gout and 709 981 matched controls. Lancet Rheumatol. 2024;6:e156–67. 38383089 10.1016/S2665-9913(23)00338-7

[18] GBD 2021 Forecasting Collaborators. Burden of disease scenarios for 204 countries and territories, 2022–2050: a forecasting analysis for the global burden of Disease Study 2021. Lancet. 2024;403:2204–56. 38762325 10.1016/S0140-6736(24)00685-8 PMC11121021

[19] GBD 2021 Causes of Death Collaborators. Global burden of 288 causes of death and life expectancy decomposition in 204 countries and territories and 811 subnational locations, 1990–2021: a systematic analysis for the global burden of Disease Study 2021. Lancet. 2024;403:2100–32. 38582094 10.1016/S0140-6736(24)00367-2 PMC11126520

[20] GBD 2021 Diseases and Injuries Collaborators. Global incidence, prevalence, years lived with disability (YLDs), disability-adjusted life-years (DALYs), and healthy life expectancy (HALE) for 371 diseases and injuries in 204 countries and territories and 811 subnational locations, 1990–2021: a systematic analysis for the global burden of Disease Study 2021. Lancet. 2024;403:2133–61. 38642570 10.1016/S0140-6736(24)00757-8 PMC11122111

[21] Laurian N, Sadov R, Strauss M, Kessler E. Laryngeal carcinoma in childhood. Report of a case and review of the literature. Laryngoscope. 1984;94:684–7. 6201689

[22] GBD 2021 Risk Factors Collaborators. Global burden and strength of evidence for 88 risk factors in 204 countries and 811 subnational locations, 1990–2021: a systematic analysis for the global burden of Disease Study 2021. Lancet. 2024;403:2162–203. 38762324 10.1016/S0140-6736(24)00933-4 PMC11120204

[23] Peller M, Katalinic A, Wollenberg B, Teudt IU, Meyer J-E. Epidemiology of laryngeal carcinoma in Germany, 1998–2011. Eur Arch Otorhinolaryngol. 2016;273:1481–7. 26879991 10.1007/s00405-016-3922-8

[24] Bikdeli B, Khairani CD, Bejjani A, Lo Y-C, Mahajan S, Caraballo C, et al. Validating International classification of diseases Code 10th revision algorithms for accurate identification of pulmonary embolism. J Thromb Haemost. 2024;S1538–7836(24):00620–2. 10.1016/j.jtha.2024.10.013 PMC12945443 39505153

[25] Cathcart-Rake EJ, Chan A, Menendez A, Markstrom D, Schnitzlein C, Chong YW et al. Cancer care for transgender and gender-diverse people: practical, literature-driven recommendations from the Multinational Association of Supportive Care in Cancer. CA Cancer J Clin. 2025;75(1):68–81. 10.3322/caac.21872 PMC11745211 39652385

[26] GBD 2021 US Burden of Disease Collaborators. The burden of diseases, injuries, and risk factors by state in the USA, 1990–2021: a systematic analysis for the global burden of Disease Study 2021. Lancet. 2024;404:2314–40. 39645376 10.1016/S0140-6736(24)01446-6 PMC11694014

[27] Jun S, Park H, Kim U-J, Lee HA, Park B, Lee SY, et al. The Combined effects of Alcohol Consumption and Smoking on Cancer risk by exposure level: a systematic review and Meta-analysis. J Korean Med Sci. 2024;39:e185. 38859742 10.3346/jkms.2024.39.e185 PMC11164648

[28] Choi SY, Kahyo H. Effect of cigarette smoking and alcohol consumption in the aetiology of cancer of the oral cavity, pharynx and larynx. Int J Epidemiol. 1991;20:878–85. 1800426 10.1093/ije/20.4.878

[29] Luo Q, Steinberg J, Yu XQ, Weber M, Caruana M, Yap S, et al. Projections of smoking-related cancer mortality in Australia to 2044. J Epidemiol Community Health. 2022;76:792–9. 35750482 10.1136/jech-2021-218252 PMC9380484

[30] di Martino E, Smith L, Bradley SH, Hemphill S, Wright J, Renzi C, et al. Incidence trends for twelve cancers in younger adults-a rapid review. Br J Cancer. 2022;126:1374–86. 35132237 10.1038/s41416-022-01704-x PMC9090760

[31] Gripp S, Pape H, Schmitt G. Chondrosarcoma of the larynx: the role of radiotherapy revisited–a case report and review of the literature. Cancer. 1998;82:108–15. 9428486 10.1002/(sici)1097-0142(19980101)82:1<108::aid-cncr13>3.0.co;2-6

[32] Tang Z-X, Gong J-L, Wang Y-H, Li Z-H, He Y, Liu Y-X, et al. Efficacy comparison between primary total laryngectomy and nonsurgical organ-preservation strategies in treatment of advanced stage laryngeal cancer: a meta-analysis. Med (Baltim). 2018;97:e10625. 10.1097/MD.0000000000010625 PMC6392597 29794737

[33] GBD 2019 Lip, Oral, and Pharyngeal Cancer Collaborators, Cunha AR da, Compton K, Xu R, Mishra R, Drangsholt MT, et al. The Global, Regional, and National Burden of Adult Lip, oral, and pharyngeal Cancer in 204 countries and territories: a systematic analysis for the global burden of Disease Study 2019. JAMA Oncol. 2023;9:1401–16. 10.1001/jamaoncol.2023.2960 PMC10485745 37676656

[34] Fan KM, Rimal J, Zhang P, Johnson NW. Stark differences in cancer epidemiological data between GLOBOCAN and GBD: emphasis on oral cancer and wider implications. EClinicalMedicine. 2022;54:101673. 36247925 10.1016/j.eclinm.2022.101673 PMC9561675

[35] Du M, Nair R, Jamieson L, Liu Z, Bi P. Incidence trends of lip, oral cavity, and pharyngeal cancers: global burden of Disease 1990–2017. J Dent Res. 2020;99:143–51. 31874128 10.1177/0022034519894963

[36] O’Sullivan A, Kabir Z, Harding M, Lip. Oral cavity and pharyngeal Cancer Burden in the European Union from 1990–2019 using the 2019 global burden of Disease Study. Int J Environ Res Public Health. 2022;19:6532. 35682117 10.3390/ijerph19116532 PMC9180496

[37] Zumsteg ZS, Cook-Wiens G, Yoshida E, Shiao SL, Lee NY, Mita A, et al. Incidence of Oropharyngeal Cancer among Elderly patients in the United States. JAMA Oncol. 2016;2:1617–23. 27415639 10.1001/jamaoncol.2016.1804

[38] Chen X, Gao L, Sturgis EM, Liang Z, Zhu Y, Xia X, et al. HPV16 DNA and integration in normal and malignant epithelium: implications for the etiology of laryngeal squamous cell carcinoma. Ann Oncol. 2017;28:1105–10. 28327951 10.1093/annonc/mdx027 PMC5406756

[39] Saraiya M, Unger ER, Thompson TD, Lynch CF, Hernandez BY, Lyu CW, et al. US assessment of HPV types in cancers: implications for current and 9-valent HPV vaccines. J Natl Cancer Inst. 2015;107:djv086. 25925419 10.1093/jnci/djv086 PMC4838063

[40] Cutts FT, Franceschi S, Goldie S, Castellsague X, de Sanjose S, Garnett G, et al. Human papillomavirus and HPV vaccines: a review. Bull World Health Organ. 2007;85:719–26. 18026629 10.2471/BLT.06.038414 PMC2636411

[41] Fountzilas E, Markou K, Vlachtsis K, Nikolaou A, Arapantoni-Dadioti P, Ntoula E, et al. Identification and validation of gene expression models that predict clinical outcome in patients with early-stage laryngeal cancer. Ann Oncol. 2012;23:2146–53. 22219018 10.1093/annonc/mdr576 PMC3493135

[42] Reis EM, Ojopi EPB, Alberto FL, Rahal P, Tsukumo F, Mancini UM, et al. Large-scale transcriptome analyses reveal new genetic marker candidates of head, neck, and thyroid cancer. Cancer Res. 2005;65:1693–9. 15753364 10.1158/0008-5472.CAN-04-3506

[43] Agalliu I, Gapstur S, Chen Z, Wang T, Anderson RL, Teras L, et al. Associations of oral α-, β-, and γ-Human papillomavirus types with risk of Incident Head and Neck Cancer. JAMA Oncol. 2016;2:599–606. 26794505 10.1001/jamaoncol.2015.5504 PMC4956584

[44] Carioli G, Bertuccio P, Levi F, Boffetta P, Negri E, La Vecchia C, et al. Cohort analysis of Epithelial Cancer Mortality Male-to-female sex ratios in the European Union, USA, and Japan. Int J Environ Res Public Health. 2020;17:5311. 32718003 10.3390/ijerph17155311 PMC7432705

[45] Nel AE, Pavlisko EN, Roggli VL. The interplay between the Immune System, Tumor suppressor genes, and Immune Senescence in Mesothelioma Development and Response to Immunotherapy. J Thorac Oncol. 2024;19:551–64. 38000500 10.1016/j.jtho.2023.11.017

[46] Bode AM, Dong Z, Wang H. Cancer prevention and control: alarming challenges in China. Natl Sci Rev. 2016;3:117–27. 27308066 10.1093/nsr/nwv054 PMC4904843

[47] Zhang N, Wu J, Wang Q, Liang Y, Li X, Chen G et al. Global burden of hematologic malignancies and evolution patterns over the past 30 years. Blood Cancer J. 2023 [cited 2023 May 24];13:82. Available from: https://www.nature.com/articles/s41408-023-00853-3 10.1038/s41408-023-00853-3 PMC10188596 37193689

[48] GBD 2019 Demographics Collaborators. Global age-sex-specific fertility, mortality, healthy life expectancy (HALE), and population estimates in 204 countries and territories, 1950–2019: a comprehensive demographic analysis for the global burden of Disease Study 2019. Lancet. 2020;396:1160–203. 33069325 10.1016/S0140-6736(20)30977-6 PMC7566045

