# Supplementary material for: Global, regional, and national mortality of larynx cancer from 1990 to 2021: results from the global burden of disease study
Source: World J Surg Oncol. 2025 Mar 7;23:76. doi: 10.1186/s12957-025-03720-6 (PMC11887237; doi:10.1186/s12957-025-03720-6)
Supplement: Supplementary file 1 — Supplementary Material 1 [file 12957_2025_3720_MOESM1_ESM.pdf]

Supplementary Table S1. Deaths and trends of larynx cancer across 204 countries from 1990 to 2021.

**Countries**	**Cases_1990**	**Rates_1990**	**Cases_2021**	**Rates_2021**	**Cases_change**	**EAPC_CI**
Afghanistan	224.21(122.97 to 345.18)	2.25(1.24 to 3.47)	266.31(145.06 to 405.31)	0.85(0.46 to 1.3)	18.77(-15.71 to 66.96)	-3.27%(-3.45 to -3.09)
Albania	66.53(53.3 to 81.72)	2.01(1.61 to 2.47)	89.77(62.38 to 128.67)	3.36(2.34 to 4.82)	34.93(-12.18 to 99.27)	2.12%(1.92 to 2.32)
Algeria	212.32(162.39 to 281.74)	0.84(0.64 to 1.11)	395.55(291.36 to 526.25)	0.89(0.66 to 1.19)	86.3(22.76 to 165.35)	0.25%(0.05 to 0.45)
American Samoa	0.22(0.17 to 0.28)	0.46(0.34 to 0.58)	0.24(0.19 to 0.31)	0.48(0.38 to 0.62)	8.06(-21.1 to 62.73)	-0.48%(-0.77 to -0.18)
Andorra	0.79(0.54 to 1.19)	1.45(0.99 to 2.18)	1.01(0.63 to 1.51)	1.18(0.74 to 1.76)	27.84(-29.9 to 123.27)	-0.63%(-0.9 to -0.36)
Angola	75.31(50.91 to 105.54)	0.73(0.5 to 1.03)	176.59(132.81 to 230.15)	0.54(0.41 to 0.7)	134.49(53.18 to 281.69)	-0.96%(-1.18 to -0.73)
Antigua and Barbuda	0.93(0.84 to 1.01)	1.54(1.4 to 1.67)	1.67(1.49 to 1.88)	1.87(1.67 to 2.1)	79.95(57.63 to 108.7)	0.76%(0.28 to 1.25)
Argentina	1094.74(1009.37 to 1198.14)	3.31(3.05 to 3.62)	891.52(805.87 to 977.75)	1.96(1.77 to 2.15)	-18.56(-28.6 to -6.97)	-1.7%(-1.85 to -1.56)
Armenia	116.82(110.97 to 121.61)	3.42(3.24 to 3.56)	74.94(66.91 to 84.09)	2.5(2.23 to 2.81)	-35.85(-43.19 to -27.35)	-1.12%(-1.4 to -0.85)
Australia	242.08(220.19 to 268.27)	1.44(1.31 to 1.59)	234.11(204.76 to 262.32)	0.91(0.79 to 1.02)	-3.29(-15.02 to 11.95)	-1.6%(-1.69 to -1.5)
Austria	209.58(193.84 to 228.24)	2.7(2.5 to 2.94)	143.56(126.13 to 159.68)	1.6(1.4 to 1.78)	-31.5(-40.86 to -21.72)	-1.51%(-1.76 to -1.27)
Azerbaijan	156.73(136.08 to 181.26)	2.14(1.86 to 2.47)	191.5(141.47 to 272.97)	1.82(1.35 to 2.6)	22.19(-9.69 to 67.66)	-0.67%(-0.8 to -0.53)
Bahamas	3.95(3.58 to 4.32)	1.54(1.39 to 1.68)	9.76(7.78 to 12.12)	2.52(2.01 to 3.12)	147.05(93.64 to 215.19)	1.81%(1.65 to 1.98)
Bahrain	4.67(3.79 to 5.91)	0.92(0.75 to 1.17)	10.34(7.44 to 15.04)	0.68(0.49 to 0.98)	121.16(58.99 to 206.76)	-2.22%(-2.69 to -1.75)
Bangladesh	1806.24(1410.68 to 2264.7)	1.66(1.29 to 2.08)	2971.74(2134.87 to 4039.15)	1.81(1.3 to 2.45)	64.53(11.08 to 135.15)	0.46%(0.26 to 0.67)
Barbados	4.13(3.77 to 4.55)	1.63(1.49 to 1.79)	7.79(6.17 to 9.74)	2.61(2.06 to 3.26)	88.63(43.53 to 137.98)	1.44%(1.21 to 1.68)
Belarus	455.4(398.82	4.36(3.82	347.3(261.96	3.72(2.8	-23.74(-42.	-1.71%(-2.

	to 523.67)	to 5.01)	to 443.52)	1 to 4.76)	25 to -0.32)	11 to -1.32)
Belgium	407.11(377.47 to 438.87)	4.08(3.78 to 4.4)	220.62(195.57 to 244.17)	1.92(1.71 to 2.13)	-45.81(-51.9 to -38.78)	-2.45%(-2.58 to -2.31)
Belize	1.14(1.06 to 1.22)	0.61(0.56 to 0.66)	4.33(3.71 to 5.02)	1.01(0.86 to 1.17)	280.08(216.16 to 350.37)	1.73%(1.28 to 2.18)
Benin	18.66(14.79 to 22.87)	0.38(0.3 to 0.47)	49.89(36.61 to 65.53)	0.37(0.27 to 0.49)	167.42(89.97 to 285.94)	0.14%(-0.02 to 0.29)
Bermuda	1.47(1.34 to 1.61)	2.47(2.26 to 2.71)	1.87(1.55 to 2.32)	2.95(2.44 to 3.66)	27.69(4.17 to 59.43)	1%(0.73 to 1.28)
Bhutan	6.11(3.98 to 8.85)	0.97(0.63 to 1.4)	11.25(7.76 to 15.52)	1.49(1.02 to 2.05)	84(22.19 to 180.33)	1.19%(1.09 to 1.29)
Bolivia (Plurinational State of)	49.54(35.63 to 65.19)	0.78(0.56 to 1.02)	93.25(64.05 to 131.43)	0.79(0.54 to 1.11)	88.24(24.13 to 190.74)	0.23%(0.1 to 0.37)
Bosnia and Herzegovina	172.56(147.48 to 200.24)	3.84(3.28 to 4.45)	164.81(117.5 to 212.62)	4.99(3.56 to 6.44)	-4.49(-33.94 to 28.61)	0.49%(0.31 to 0.68)
Botswana	14.4(10.54 to 18.41)	1.09(0.8 to 1.4)	25.58(17.92 to 39.81)	1.07(0.75 to 1.66)	77.65(16.48 to 183.04)	-0.51%(-0.78 to -0.25)
Brazil	2570.46(2463.04 to 2672.4)	1.73(1.66 to 1.8)	5497.44(5144.06 to 5811.97)	2.49(2.33 to 2.64)	113.87(101.42 to 126.66)	1.26%(1.17 to 1.35)
Brunei Darussalam	1.89(1.47 to 2.37)	0.73(0.57 to 0.91)	2.4(1.88 to 3.08)	0.53(0.42 to 0.68)	27.25(-7.47 to 87.48)	-0.99%(-1.32 to -0.66)
Bulgaria	376.15(322.33 to 437.56)	4.33(3.71 to 5.04)	420.63(335.54 to 518.13)	6.2(4.94 to 7.63)	11.82(-15.85 to 46.73)	1.5%(1.28 to 1.73)
Burkina Faso	41.24(30.8 to 53.42)	0.43(0.32 to 0.56)	103.51(72.55 to 140.67)	0.45(0.32 to 0.62)	150.99(76.14 to 261.58)	0.55%(0.34 to 0.75)
Burundi	53.19(34.2 to 74.08)	0.96(0.62 to 1.33)	70.05(48.6 to 101.01)	0.53(0.37 to 0.76)	31.7(-6.59 to 92.75)	-2.37%(-2.72 to -2.03)
Cabo Verde	3.57(2.83 to 4.46)	1.01(0.8 to 1.26)	6.31(4.31 to 8.06)	1.13(0.77 to 1.44)	76.49(12.96 to 153.21)	0.08%(-0.61 to 0.77)
Cambodia	79.6(59.47 to 106.74)	0.77(0.58 to 1.04)	181.24(125.59 to 272.1)	1.06(0.74 to 1.6)	127.68(51.54 to 274.36)	1.11%(0.87 to 1.34)
Cameroon	45.16(35.38 to 58.63)	0.43(0.34 to 0.56)	151.48(99.25 to 218.97)	0.48(0.31 to 0.69)	235.4(109.5 to 395)	0.5%(0.4 to 0.59)
Canada	485.43(447.4	1.78(1.64	435.96(389.22	1.16(1.0	-10.19(-21.	-1.2%(-1.3

	to 524.96)	to 1.93)	to 483.78)	4 to 1.29)	24 to 2.64)	1 to -1.09)
Central African Republic	25.41(15.22 to 35.09)	0.93(0.56 to 1.28)	39.35(23.36 to 57.4)	0.72(0.43 to 1.05)	54.88(11.58 to 111.35)	-0.89%(-1.13 to -0.65)
Chad	22.19(17.07 to 28.54)	0.37(0.28 to 0.47)	74.13(52.14 to 99.34)	0.42(0.29 to 0.56)	234.11(123.88 to 366)	0.69%(0.56 to 0.81)
Chile	147.47(133.88 to 163.7)	1.11(1.01 to 1.23)	153.38(135.61 to 170.96)	0.82(0.72 to 0.91)	4.01(-10.07 to 20.94)	-0.79%(-0.92 to -0.65)
China	12869.79(10565.15 to 15142.78)	1.09(0.9 to 1.29)	19799.45(15579.57 to 25023.24)	1.39(1.1 to 1.76)	53.84(15.12 to 102.68)	0.79%(0.63 to 0.94)
Colombia	416.31(382.59 to 447.35)	1.28(1.18 to 1.38)	482.11(392.34 to 584.61)	0.98(0.8 to 1.19)	15.81(-6.41 to 42.41)	-1.39%(-1.56 to -1.23)
Comoros	3.65(2.44 to 4.88)	0.79(0.53 to 1.06)	6.44(4.68 to 9)	0.87(0.63 to 1.21)	76.61(17.89 to 172.13)	0%(-0.25 to 0.24)
Congo	22.32(14.26 to 29.27)	0.93(0.59 to 1.22)	41.93(30.8 to 55.84)	0.78(0.57 to 1.04)	87.9(33.83 to 209.37)	-0.86%(-1.16 to -0.55)
Cook Islands	0.06(0.05 to 0.08)	0.34(0.26 to 0.41)	0.09(0.07 to 0.12)	0.5(0.38 to 0.67)	38.89(-4.45 to 114.49)	1.21%(0.86 to 1.56)
Costa Rica	29.48(26.65 to 32.28)	0.97(0.88 to 1.06)	39.31(33.93 to 45.91)	0.83(0.71 to 0.97)	33.32(11.22 to 58.73)	-0.48%(-0.64 to -0.33)
Côte d'Ivoire	51.8(40.44 to 66.61)	0.42(0.33 to 0.55)	126.67(87.51 to 195.5)	0.45(0.31 to 0.7)	144.52(70.54 to 265.52)	-0.1%(-0.27 to 0.08)
Croatia	283.42(256.06 to 312.18)	5.83(5.27 to 6.42)	177.47(149.62 to 206.57)	4.22(3.55 to 4.91)	-37.38(-48.21 to -24.98)	-1.13%(-1.3 to -0.96)
Cuba	431.22(389.45 to 480.29)	3.98(3.59 to 4.43)	921.23(780.07 to 1113.76)	8.17(6.92 to 9.88)	113.63(75.08 to 164.48)	2.51%(2.38 to 2.63)
Cyprus	11.52(9.21 to 14.4)	1.48(1.18 to 1.85)	15.34(12.21 to 19.66)	1.13(0.9 to 1.45)	33.2(-2.05 to 84.65)	-0.85%(-0.91 to -0.78)
Czechia	349.66(306.56 to 400.84)	3.4(2.98 to 3.89)	244.99(195.16 to 303.34)	2.3(1.84 to 2.85)	-29.94(-44.87 to -6.56)	-1.03%(-1.14 to -0.92)
Democratic People's Republic of Korea	160.96(114.88 to 216.89)	0.78(0.56 to 1.05)	278.3(199.23 to 367.64)	1.05(0.75 to 1.39)	72.9(18.79 to 145.56)	0.91%(0.87 to 0.95)
Democratic Republic of the Congo	231.79(161.14 to 312.8)	0.61(0.42 to 0.82)	477.59(329.83 to 639.95)	0.53(0.37 to 0.71)	106.04(33.95 to 198.68)	-0.52%(-0.76 to -0.28)
Denmark	150.31(138.59	2.92(2.69	104.24(93.54	1.78(1.6	-30.65(-38.	-1.84%(-2.

	to 163.24)	to 3.17)	to 115.55)	to 1.97)	98 to -22.1)	03 to -1.66)
Djibouti	2.85(1.95 to 4.33)	0.69(0.47 to 1.04)	11.34(7.66 to 16.04)	0.9(0.61 to 1.27)	298.24(158.92 to 496.22)	0.78%(0.64 to 0.92)
Dominica	1.3(1.09 to 1.57)	1.8(1.5 to 2.16)	1.99(1.49 to 2.58)	2.96(2.23 to 3.85)	52.31(10.02 to 110.44)	1.67%(1.51 to 1.84)
Dominican Republic	58.16(46.16 to 73.22)	0.81(0.65 to 1.02)	153.62(111.09 to 206.12)	1.39(1.01 to 1.87)	164.14(69.01 to 286.37)	2.08%(1.93 to 2.23)
Ecuador	53.6(49.51 to 58.25)	0.54(0.5 to 0.58)	87.34(68.44 to 111.73)	0.48(0.38 to 0.62)	62.95(24.19 to 112.1)	-0.19%(-0.47 to 0.1)
Egypt	299.41(252.6 to 380.2)	0.54(0.46 to 0.69)	647.42(498.75 to 835.62)	0.61(0.47 to 0.79)	116.23(59.48 to 181.83)	0.62%(0.46 to 0.77)
El Salvador	27.89(23.79 to 32.63)	0.53(0.45 to 0.61)	41.74(32.55 to 53.22)	0.65(0.5 to 0.83)	49.66(9.11 to 104.71)	0.39%(0.2 to 0.57)
Equatorial Guinea	3.78(2.3 to 5.45)	0.89(0.54 to 1.29)	6(3.55 to 8.84)	0.4(0.23 to 0.58)	58.9(-4.85 to 208.54)	-2.77%(-3.32 to -2.22)
Eritrea	26.63(18.41 to 36.94)	0.78(0.54 to 1.08)	45.92(31.53 to 63.18)	0.7(0.48 to 0.96)	72.46(23.93 to 146.96)	-0.66%(-0.8 to -0.53)
Estonia	51.44(45.62 to 57.96)	3.28(2.91 to 3.7)	29.38(23.99 to 35.19)	2.24(1.83 to 2.68)	-42.89(-55.31 to -29.11)	-1.91%(-2.24 to -1.59)
Eswatini	8.26(5.28 to 11.38)	1.02(0.65 to 1.41)	15.22(9.01 to 22.11)	1.32(0.78 to 1.91)	84.34(18.27 to 185.05)	1.11%(0.67 to 1.56)
Ethiopia	323.95(180.34 to 467.51)	0.64(0.36 to 0.92)	387.31(276.28 to 521.67)	0.36(0.25 to 0.48)	19.56(-16.88 to 102.48)	-2.12%(-2.4 to -1.84)
Fiji	2.37(1.85 to 3.04)	0.31(0.24 to 0.4)	4.85(3.5 to 6.39)	0.52(0.38 to 0.69)	104.39(41.5 to 188.07)	1.96%(1.77 to 2.14)
Finland	56.09(51.17 to 61.06)	1.12(1.02 to 1.22)	49.09(43.48 to 54.74)	0.89(0.79 to 0.99)	-12.49(-23.42 to -0.56)	-0.79%(-1.09 to -0.49)
France	3003.9(2755.65 to 3282.36)	5.2(4.77 to 5.68)	1667.14(1464.98 to 1868.91)	2.51(2.21 to 2.82)	-44.5(-52.29 to -36.25)	-2.23%(-2.63 to -1.82)
Gabon	11.47(8.43 to 15.07)	1.17(0.86 to 1.53)	16.7(12 to 22.3)	0.92(0.66 to 1.23)	45.6(3.21 to 105.42)	-0.82%(-0.97 to -0.68)
Gambia	1.74(1.33 to 2.27)	0.18(0.14 to 0.23)	4.47(3.32 to 5.79)	0.19(0.14 to 0.24)	157.06(80.02 to 267.97)	0.08%(-0.08 to 0.25)
Georgia	256.62(235.86	4.65(4.27	182.24(157.54	5.05(4.3	-28.99(-39.	0.74%(0.45

	to 277.95)	to 5.03)	to 207.67)	7 to 5.76)	76 to -18.32)	to 1.03)
Germany	1947.5(1802.25 to 2111)	2.44(2.25 to 2.64)	1562.26(1402.14 to 1732.83)	1.83(1.64 to 2.03)	-19.78(-29.01 to -7.65)	-0.88%(-0.99 to -0.76)
Ghana	48.76(37.75 to 64.39)	0.33(0.25 to 0.43)	176.35(125.15 to 230.75)	0.51(0.37 to 0.67)	261.69(148.44 to 414.61)	2.03%(1.79 to 2.27)
Greece	420.36(394.26 to 444.06)	4.05(3.79 to 4.27)	384.26(348.58 to 414.88)	3.78(3.43 to 4.08)	-8.59(-16.78 to -0.25)	-0.06%(-0.2 to 0.07)
Greenland	0.91(0.73 to 1.11)	1.64(1.31 to 2)	0.85(0.67 to 1.1)	1.52(1.19 to 1.97)	-6.42(-31.53 to 31.39)	0.2%(-0.06 to 0.46)
Grenada	1.07(0.95 to 1.21)	1.23(1.09 to 1.39)	1.34(1.13 to 1.57)	1.3(1.1 to 1.53)	24.76(3.19 to 50.56)	0.85%(0.5 to 1.2)
Guam	0.37(0.31 to 0.43)	0.27(0.23 to 0.31)	0.5(0.42 to 0.58)	0.31(0.26 to 0.36)	33.25(7.52 to 73.49)	0.35%(0.04 to 0.67)
Guatemala	51.29(48.95 to 53.88)	0.61(0.58 to 0.64)	52.57(44.93 to 61.56)	0.33(0.28 to 0.39)	2.5(-13.24 to 20.1)	-1.98%(-2.21 to -1.74)
Guinea	27.08(20.67 to 34.75)	0.45(0.34 to 0.58)	67.12(49.1 to 86.55)	0.5(0.37 to 0.64)	147.8(65.48 to 255.77)	0.83%(0.6 to 1.06)
Guinea-Bissau	5.53(3.49 to 7.81)	0.55(0.35 to 0.78)	10.89(7.28 to 14.92)	0.53(0.35 to 0.72)	96.94(32.83 to 192.07)	0.33%(0.16 to 0.51)
Guyana	4.48(3.89 to 5.21)	0.57(0.5 to 0.67)	7.1(5.28 to 9.33)	0.93(0.69 to 1.22)	58.47(10.39 to 116.15)	2.43%(2.2 to 2.67)
Haiti	90.44(61.1 to 119.69)	1.42(0.96 to 1.88)	159.43(102.76 to 224.26)	1.24(0.8 to 1.74)	76.29(15.74 to 164.35)	-0.29%(-0.44 to -0.13)
Honduras	23.04(18.94 to 28.75)	0.49(0.4 to 0.61)	86.81(64.39 to 111.85)	0.86(0.64 to 1.11)	276.74(164.6 to 411.56)	2.15%(2.01 to 2.29)
Hungary	629.49(563.32 to 696.73)	6.06(5.42 to 6.7)	482.71(401.65 to 574.82)	5.03(4.19 to 5.99)	-23.32(-36.7 to -6.14)	-0.87%(-0.99 to -0.74)
Iceland	2.26(2.03 to 2.48)	0.89(0.8 to 0.98)	2.07(1.79 to 2.34)	0.59(0.51 to 0.67)	-8.33(-20.95 to 5.7)	-1.45%(-1.67 to -1.24)
India	14101.91(11465.35 to 16898.08)	1.65(1.34 to 1.98)	28330.04(24663.74 to 32828.51)	2(1.74 to 2.32)	100.9(57.89 to 149.21)	0.5%(0.3 to 0.71)
Indonesia	1032.34(773.63 to 1253.41)	0.56(0.42 to 0.68)	2366.43(1668.49 to 3118.31)	0.85(0.6 to 1.12)	129.23(70.6 to 200.88)	1.25%(1.21 to 1.29)
Iran (Islamic Republic of)	673.86(562.6 to 766.48)	1.18(0.99 to 1.34)	1345.99(1193.35 to 1501.56)	1.58(1.4 to 1.76)	99.74(69.91 to 146.73)	1.19%(1.09 to 1.29)

Iraq	193.78(148.26 to 246.59)	1.05(0.81 to 1.34)	463.14(329.58 to 617.52)	1.12(0.8 to 1.5)	139(56.35 to 246.84)	-0.06%(-0.23 to 0.12)
Ireland	72.36(65.81 to 79.37)	2.01(1.83 to 2.2)	52.06(45.95 to 58.3)	1.05(0.93 to 1.18)	-28.05(-37.4 to -17.46)	-1.97%(-2.22 to -1.72)
Israel	57.47(51.85 to 64.05)	1.16(1.05 to 1.29)	83.42(73.16 to 94.84)	0.87(0.76 to 0.99)	45.16(24.07 to 72.48)	-1.39%(-1.61 to -1.18)
Italy	2599.43(2475.32 to 2720.74)	4.58(4.36 to 4.79)	1641.07(1486.56 to 1774.47)	2.74(2.49 to 2.97)	-36.87(-42.45 to -31.37)	-1.75%(-1.89 to -1.61)
Jamaica	20.83(18.58 to 23.12)	0.88(0.79 to 0.98)	39.33(28.55 to 52.29)	1.4(1.02 to 1.87)	88.77(34.73 to 153.88)	1.65%(1.24 to 2.06)
Japan	981.14(933.73 to 1012.55)	0.78(0.74 to 0.8)	1092.46(966.58 to 1164.8)	0.86(0.76 to 0.91)	11.35(2.36 to 17.11)	0.06%(-0.1 to 0.22)
Jordan	19.75(15.08 to 25.36)	0.53(0.4 to 0.68)	48.65(34.6 to 67.59)	0.39(0.28 to 0.55)	146.36(64.62 to 274.21)	-1.8%(-2.14 to -1.46)
Kazakhstan	426.85(393.26 to 459.78)	2.6(2.4 to 2.8)	221.54(190.58 to 253.59)	1.17(1.01 to 1.34)	-48.1(-55.73 to -38.9)	-3.37%(-3.69 to -3.06)
Kenya	95.11(69.03 to 129.38)	0.41(0.3 to 0.56)	304.59(224.39 to 393.74)	0.61(0.45 to 0.79)	220.26(146.19 to 324.57)	1.34%(1.23 to 1.45)
Kiribati	0.07(0.06 to 0.09)	0.1(0.08 to 0.13)	0.15(0.11 to 0.21)	0.12(0.09 to 0.18)	104.05(46.8 to 184.25)	0.71%(0.68 to 0.74)
Kuwait	9.12(8.06 to 10.16)	0.53(0.47 to 0.59)	10.38(8.47 to 12.92)	0.22(0.18 to 0.28)	13.84(-10.59 to 43.27)	-2.53%(-3.13 to -1.93)
Kyrgyzstan	61.32(51.46 to 72.41)	1.37(1.15 to 1.62)	37.7(28.18 to 47.86)	0.55(0.41 to 0.7)	-38.53(-54.57 to -18.67)	-3.06%(-3.23 to -2.9)
Lao People's Democratic Republic	36.46(25.64 to 50.49)	0.87(0.62 to 1.21)	50.95(34.87 to 74.74)	0.69(0.47 to 1.01)	39.75(-11.93 to 124.68)	-0.8%(-1.08 to -0.52)
Latvia	110.01(99.02 to 123.08)	4.14(3.73 to 4.63)	61.87(51.47 to 74.13)	3.31(2.75 to 3.96)	-43.76(-54.58 to -30.18)	-0.91%(-1.1 to -0.72)
Lebanon	63.57(43.99 to 85.29)	2.12(1.47 to 2.85)	110.8(89.18 to 137.53)	2(1.61 to 2.48)	74.29(19.33 to 168)	0.11%(-0.22 to 0.44)
Lesotho	17.25(13.04 to 22.31)	1.13(0.85 to 1.46)	35.2(23.92 to 48.27)	1.88(1.28 to 2.58)	103.99(46.01 to 190.94)	2.1%(1.71 to 2.5)
Liberia	10.24(7.39 to 14.76)	0.42(0.3 to 0.6)	20.63(13.92 to 29.55)	0.38(0.25 to 0.54)	101.52(33.79 to 206.18)	-1.01%(-1.34 to -0.68)
Libya	59.69(43.54 to	1.42(1.03	157.8(113.4 to	2.3(1.65	164.37(68.1	1.59%(1.36

	82.03)	to 1.95)	215.13)	to 3.13)	8 to 288.31)	to 1.82)
Lithuania	152.63(133.2 to 174.67)	4.15(3.63 to 4.75)	110.75(87.64 to 135.6)	4.06(3.21 to 4.97)	-27.44(-44.63 to -8.56)	-0.16%(-0.4 to 0.08)
Luxembourg	13.94(12.88 to 15.03)	3.66(3.38 to 3.94)	9.84(8.75 to 10.97)	1.53(1.36 to 1.7)	-29.4(-38.3 to -19.45)	-2.73%(-2.96 to -2.51)
Madagascar	82.14(60.56 to 102.99)	0.69(0.51 to 0.87)	123.79(87.23 to 166.72)	0.43(0.31 to 0.58)	50.72(7.96 to 100.68)	-1.53%(-1.83 to -1.22)
Malawi	22.59(17.31 to 30.16)	0.23(0.18 to 0.31)	43.98(33.05 to 61.45)	0.23(0.17 to 0.32)	94.7(46.3 to 164.19)	-0.46%(-0.68 to -0.23)
Malaysia	140.86(107.43 to 174.51)	0.8(0.61 to 0.99)	345.83(272.05 to 415.5)	1.09(0.86 to 1.31)	145.52(77.4 to 217.93)	0.96%(0.6 to 1.33)
Maldives	0.99(0.71 to 1.27)	0.45(0.32 to 0.57)	1.51(1.12 to 1.96)	0.29(0.22 to 0.38)	51.71(2.2 to 135.9)	-1.71%(-1.8 to -1.61)
Mali	42.23(35.12 to 51.11)	0.49(0.41 to 0.59)	84.32(62 to 116.12)	0.35(0.26 to 0.48)	99.68(40.37 to 181.88)	-0.75%(-0.93 to -0.56)
Malta	8.47(7.41 to 9.57)	2.29(2 to 2.58)	7.08(6.04 to 8.23)	1.6(1.37 to 1.86)	-16.42(-30.74 to 0.38)	-1.18%(-1.27 to -1.1)
Marshall Islands	0.12(0.09 to 0.17)	0.27(0.19 to 0.37)	0.23(0.15 to 0.33)	0.41(0.27 to 0.59)	91.38(37.24 to 180.84)	1.64%(1.48 to 1.8)
Mauritania	8.93(6.97 to 11.29)	0.43(0.34 to 0.55)	19.91(13.9 to 27.91)	0.45(0.32 to 0.63)	122.87(47.78 to 223.69)	0.37%(0.08 to 0.66)
Mauritius	14.82(13.9 to 15.98)	1.35(1.27 to 1.46)	27.22(24.78 to 29.31)	2.14(1.95 to 2.3)	83.72(64.44 to 102.26)	1.89%(0.99 to 2.81)
Mexico	804.09(779.97 to 825.78)	0.94(0.91 to 0.97)	971.99(830.03 to 1122.81)	0.75(0.64 to 0.87)	20.88(3.68 to 40.47)	-1.04%(-1.16 to -0.92)
Micronesia (Federated States of)	0.38(0.29 to 0.51)	0.37(0.28 to 0.49)	0.46(0.33 to 0.63)	0.45(0.32 to 0.61)	19.94(-16.32 to 69.08)	0.57%(0.39 to 0.75)
Monaco	3.51(2.53 to 4.71)	11.56(8.33 to 15.47)	2.99(2.31 to 3.87)	7.9(6.09 to 10.22)	-14.89(-42.52 to 23.42)	-1.35%(-1.47 to -1.23)
Mongolia	15.67(11.4 to 20.4)	0.73(0.53 to 0.95)	19.6(14.28 to 26.96)	0.59(0.43 to 0.81)	25.09(-17.34 to 92.09)	-0.83%(-0.94 to -0.73)
Montenegro	33.05(26.28 to 41.44)	5.28(4.2 to 6.62)	45.88(35.37 to 60.22)	7.42(5.72 to 9.74)	38.83(6.14 to 85.71)	1.02%(0.74 to 1.29)
Morocco	278.63(215.29 to 349.35)	1.1(0.85 to 1.38)	603.57(427.29 to 782.87)	1.62(1.15 to	116.62(44.86 to 205.66)	1.44%(1.16 to 1.72)

				2.11)		
Mozambique	112.83(82.83 to 150.49)	0.84(0.62 to 1.13)	210.96(153.7 to 277.06)	0.68(0.49 to 0.89)	86.98(37.64 to 158.82)	-0.36%(-0.5 to -0.21)
Myanmar	374.44(237.47 to 514.29)	0.93(0.59 to 1.27)	429.61(303.73 to 607.3)	0.76(0.54 to 1.08)	14.74(-23.6 to 75.09)	-0.89%(-1.05 to -0.72)
Namibia	16.54(13.31 to 20.37)	1.18(0.95 to 1.45)	34.88(25.43 to 47.07)	1.43(1.05 to 1.94)	110.88(53.5 to 185.87)	0.48%(0.28 to 0.68)
Nauru	0.04(0.03 to 0.06)	0.42(0.27 to 0.56)	0.04(0.03 to 0.06)	0.37(0.23 to 0.52)	-4.77(-35.37 to 43.96)	-0.65%(-0.91 to -0.4)
Nepal	277.15(190.42 to 386.13)	1.42(0.98 to 1.98)	503.24(367.49 to 707.34)	1.62(1.18 to 2.27)	81.58(22.61 to 163.21)	0.61%(0.25 to 0.97)
Netherlands	267.55(249.01 to 288.33)	1.79(1.67 to 1.93)	224.33(201.2 to 245.71)	1.3(1.17 to 1.43)	-16.16(-24.89 to -5.91)	-1.24%(-1.38 to -1.09)
New Zealand	35.36(31.99 to 38.93)	1.03(0.94 to 1.14)	32.22(27.83 to 36.5)	0.62(0.54 to 0.71)	-8.9(-22.61 to 5.77)	-1.56%(-1.91 to -1.2)
Nicaragua	17.72(14.34 to 21.5)	0.46(0.37 to 0.55)	35.51(27.65 to 46.39)	0.53(0.41 to 0.7)	100.4(45.77 to 176.61)	0.46%(0.29 to 0.62)
Niger	23.65(17.27 to 31.24)	0.29(0.22 to 0.39)	73.41(48.96 to 103.96)	0.29(0.2 to 0.42)	210.34(114.78 to 345.97)	0.43%(0.25 to 0.61)
Nigeria	608.62(424.07 to 820.34)	0.68(0.47 to 0.91)	976.51(677.12 to 1328.63)	0.42(0.29 to 0.57)	60.45(3.81 to 165.89)	-1.59%(-1.64 to -1.54)
Niue	0.01(0.01 to 0.02)	0.52(0.38 to 0.68)	0.01(0.01 to 0.01)	0.64(0.47 to 0.83)	-11.87(-34.22 to 16.86)	0.54%(0.21 to 0.88)
North Macedonia	79.85(65.56 to 99.32)	4.01(3.29 to 4.99)	108.48(82.1 to 141.24)	4.98(3.77 to 6.49)	35.86(0.18 to 79.42)	0.59%(0.35 to 0.84)
Northern Mariana Islands	0.16(0.12 to 0.22)	0.36(0.26 to 0.48)	0.3(0.24 to 0.36)	0.62(0.5 to 0.75)	83.26(32.19 to 164.9)	1.66%(1.27 to 2.06)
Norway	46.66(43.56 to 49.23)	1.1(1.03 to 1.16)	38.33(34.38 to 41.57)	0.71(0.63 to 0.77)	-17.85(-25.53 to -10)	-1.79%(-2.07 to -1.52)
Oman	5.49(3.86 to 7.61)	0.28(0.19 to 0.38)	8.51(6.39 to 11.37)	0.18(0.14 to 0.24)	54.87(0.46 to 147.71)	-1.19%(-1.37 to -1.02)
Pakistan	2817.36(2265.48 to 3485.56)	2.54(2.04 to 3.14)	5616.89(4105.95 to 7453.41)	2.38(1.74 to 3.16)	99.37(40.66 to 182.83)	-0.57%(-0.75 to -0.39)
Palau	0.05(0.04 to 0.07)	0.35(0.25 to 0.46)	0.09(0.07 to 0.12)	0.51(0.37 to	73.86(23.12 to 142.72)	1.18%(1.08 to 1.28)

				0.68)		
Palestine	11.73(8.69 to 15.33)	0.57(0.42 to 0.75)	21.42(17.17 to 26.48)	0.42(0.33 to 0.52)	82.64(28.44 to 158.11)	-1.3%(-1.42 to -1.18)
Panama	23.18(21.58 to 24.63)	0.97(0.9 to 1.03)	31.99(24.99 to 38.9)	0.75(0.58 to 0.91)	37.98(8.57 to 66.69)	-1.06%(-1.26 to -0.87)
Papua New Guinea	8.55(5.6 to 12.29)	0.21(0.14 to 0.3)	21.66(14.95 to 31.75)	0.21(0.14 to 0.3)	153.16(68.89 to 287.36)	-0.15%(-0.27 to -0.02)
Paraguay	28.84(22.48 to 37.11)	0.71(0.56 to 0.92)	95.67(66.24 to 130.92)	1.33(0.92 to 1.83)	231.72(113.4 to 405.56)	2.25%(2.17 to 2.34)
Peru	133.81(107.57 to 162.57)	0.62(0.5 to 0.75)	186.78(132.49 to 258.46)	0.51(0.37 to 0.71)	39.59(-9.58 to 109.69)	-0.8%(-1.12 to -0.48)
Philippines	269.29(226.03 to 329.5)	0.43(0.36 to 0.52)	677.77(546.06 to 816.99)	0.6(0.48 to 0.72)	151.69(90.6 to 224.17)	1.06%(1.02 to 1.09)
Poland	1838.52(1768.12 to 1911.02)	4.82(4.63 to 5.01)	1771.71(1593.69 to 1945.45)	4.63(4.17 to 5.09)	-3.63(-13.67 to 6.41)	-0.27%(-0.41 to -0.13)
Portugal	474.02(427.53 to 522.76)	4.68(4.22 to 5.16)	355.84(314.24 to 405.27)	3.35(2.96 to 3.82)	-24.93(-35.72 to -12.83)	-1.19%(-1.28 to -1.11)
Puerto Rico	83.61(74.74 to 92.31)	2.31(2.07 to 2.56)	58.86(47.53 to 71.77)	1.79(1.44 to 2.18)	-29.6(-44.75 to -12.5)	-0.67%(-0.93 to -0.41)
Qatar	2.56(1.92 to 3.38)	0.57(0.43 to 0.76)	10.53(7.29 to 14.61)	0.35(0.24 to 0.49)	312.12(182.54 to 490.82)	-2.1%(-2.8 to -1.4)
Republic of Korea	649.14(459.88 to 822.69)	1.47(1.04 to 1.86)	504.52(370.83 to 647.75)	0.98(0.72 to 1.26)	-22.28(-45.12 to 23.75)	-2.3%(-2.65 to -1.93)
Republic of Moldova	158.91(149.43 to 169.04)	3.57(3.36 to 3.8)	146.54(129.72 to 166.92)	4.08(3.61 to 4.65)	-7.78(-18.78 to 6.9)	0.48%(0.17 to 0.79)
Romania	911.51(803 to 1019.92)	3.9(3.43 to 4.36)	1049.16(894 to 1222.24)	5.54(4.72 to 6.45)	15.1(-4.1 to 40.84)	1.01%(0.8 to 1.21)
Russian Federation	6222.56(6045.66 to 6380.7)	4.12(4 to 4.23)	4013.73(3572.38 to 4418.24)	2.77(2.47 to 3.05)	-35.5(-42.99 to -28.97)	-2.02%(-2.28 to -1.77)
Rwanda	76.64(54.37 to 99.31)	1.07(0.76 to 1.38)	93.13(62.87 to 137.93)	0.7(0.47 to 1.04)	21.53(-17.42 to 82.82)	-2.2%(-2.71 to -1.7)
Saint Kitts and Nevis	0.59(0.54 to 0.65)	1.43(1.31 to 1.56)	1.11(0.9 to 1.34)	1.9(1.54 to 2.29)	87.1(50.83 to 131.53)	1.18%(0.72 to 1.65)
Saint Lucia	2.07(1.94 to 2.22)	1.52(1.42 to 1.62)	4.99(4.05 to 6.07)	2.81(2.28 to 3.42)	140.88(96 to 194.6)	2.01%(1.69 to 2.33)

Saint Vincent and the Grenadines	1.69(1.53 to 1.86)	1.54(1.39 to 1.7)	3.85(3.35 to 4.42)	3.37(2.93 to 3.87)	127.95(92.47 to 173.1)	2.72%(2.5 to 2.94)
Samoa	0.24(0.18 to 0.31)	0.14(0.11 to 0.18)	0.35(0.28 to 0.47)	0.17(0.13 to 0.22)	48.57(11.6 to 100.44)	0.47%(0.41 to 0.53)
San Marino	0.76(0.59 to 0.95)	3.21(2.48 to 3.99)	0.6(0.37 to 0.87)	1.83(1.14 to 2.64)	-21.36(-53.13 to 18.72)	-0.96%(-1.32 to -0.61)
Sao Tome and Principe	0.43(0.33 to 0.54)	0.35(0.27 to 0.45)	0.8(0.63 to 1.03)	0.37(0.29 to 0.47)	86.08(34.98 to 155.99)	0.16%(0.02 to 0.3)
Saudi Arabia	51.53(38.4 to 67.14)	0.32(0.24 to 0.42)	118.65(89.9 to 159.68)	0.31(0.24 to 0.42)	130.27(56.12 to 258.73)	-0.64%(-0.82 to -0.47)
Senegal	32(25.29 to 39.12)	0.42(0.33 to 0.51)	86.04(62.26 to 112.77)	0.54(0.39 to 0.71)	168.86(91.99 to 272.13)	1.24%(1.02 to 1.46)
Serbia	477.45(343.4 to 674.76)	4.96(3.57 to 7.01)	449.06(310.56 to 616.69)	5.03(3.48 to 6.91)	-5.95(-40.96 to 46.65)	-0.12%(-0.37 to 0.13)
Seychelles	2.92(2.5 to 3.45)	4.01(3.43 to 4.73)	4.59(3.73 to 5.51)	4.35(3.54 to 5.23)	56.93(22.01 to 101.04)	0.9%(0.55 to 1.25)
Sierra Leone	19.58(14.67 to 25.4)	0.47(0.35 to 0.61)	39.51(28.09 to 55.71)	0.45(0.32 to 0.63)	101.82(40.39 to 194.93)	-0.08%(-0.21 to 0.06)
Singapore	32.71(29.49 to 35.92)	1.07(0.97 to 1.18)	34.71(30.04 to 39.2)	0.61(0.52 to 0.68)	6.12(-11.46 to 24.21)	-2.6%(-3.06 to -2.14)
Slovakia	230.28(174.93 to 298.79)	4.36(3.31 to 5.66)	189.22(137.13 to 250.09)	3.48(2.53 to 4.61)	-17.83(-44.13 to 15.7)	-0.69%(-0.77 to -0.61)
Slovenia	66.37(59.93 to 74.26)	3.36(3.04 to 3.76)	51.13(42.52 to 59.8)	2.47(2.05 to 2.89)	-22.95(-36.53 to -6.02)	-1.2%(-1.4 to -1.01)
Solomon Islands	1(0.6 to 1.45)	0.3(0.18 to 0.43)	2.22(1.58 to 3.05)	0.33(0.23 to 0.45)	122.02(46.78 to 264.35)	0.31%(0.23 to 0.39)
Somalia	54.26(34.83 to 79.66)	0.68(0.44 to 1)	104.53(65.02 to 153.74)	0.48(0.3 to 0.71)	92.64(38.09 to 168.8)	-1.42%(-1.57 to -1.28)
South Africa	396.81(322.26 to 540.78)	1.07(0.87 to 1.46)	746.49(654.95 to 841.64)	1.31(1.15 to 1.48)	88.12(43.88 to 135.08)	0.37%(0.1 to 0.63)
South Sudan	51.37(35.62 to 69.3)	0.87(0.61 to 1.18)	61.12(39.36 to 90.29)	0.63(0.41 to 0.93)	18.99(-22.03 to 78.52)	-1.47%(-1.99 to -0.94)
Spain	2205.72(1940.	5.69(5 to	1414.76(1242.	3.11(2.7	-35.86(-45.	-2.32%(-2.

	52 to 2459.97)	6.34)	07 to 1612.37)	3 to 3.54)	92 to -23.88)	49 to -2.14)
Sri Lanka	94.46(76.71 to 119.22)	0.55(0.45 to 0.7)	250.93(148 to 376.63)	1.13(0.66 to 1.69)	165.66(43.6 to 309.75)	3.42%(2.99 to 3.86)
Sudan	218.43(134.48 to 342.88)	1.09(0.67 to 1.71)	347.31(216.17 to 513.11)	0.8(0.5 to 1.18)	59(-6.75 to 143.2)	-1.17%(-1.28 to -1.05)
Suriname	2.24(1.84 to 2.66)	0.58(0.48 to 0.69)	5.05(3.58 to 7)	0.87(0.62 to 1.21)	125.06(50.31 to 233.85)	1.48%(1.31 to 1.65)
Sweden	73.8(67.03 to 80.38)	0.86(0.78 to 0.94)	62.82(53.83 to 72.07)	0.61(0.52 to 0.69)	-14.88(-27.77 to -1.12)	-0.98%(-1.24 to -0.73)
Switzerland	139.57(128.99 to 150.93)	2.03(1.88 to 2.2)	96.18(84.38 to 107.18)	1.08(0.95 to 1.2)	-31.09(-39.67 to -21.8)	-1.77%(-1.99 to -1.56)
Syrian Arab Republic	75.62(57.89 to 99.11)	0.59(0.46 to 0.78)	141.94(103.15 to 200.85)	1.01(0.74 to 1.43)	87.72(20.53 to 189.48)	1.35%(0.6 to 2.1)
Taiwan (Province of China)	186.11(174.55 to 200.55)	0.91(0.86 to 0.98)	250.34(224.31 to 276.11)	1.06(0.95 to 1.17)	34.51(19.93 to 50.87)	0.12%(-0.09 to 0.33)
Tajikistan	44.77(33.36 to 56.39)	0.83(0.62 to 1.05)	52.99(35.77 to 74.66)	0.52(0.35 to 0.73)	18.36(-23.39 to 80.86)	-1.65%(-1.81 to -1.48)
Thailand	694.33(564.79 to 828.62)	1.22(0.99 to 1.46)	1346.13(994.28 to 1756.76)	2.02(1.49 to 2.63)	93.88(39.4 to 171.72)	1.3%(1.14 to 1.46)
Timor-Leste	2.75(1.88 to 3.95)	0.35(0.24 to 0.51)	7.8(5.53 to 10.96)	0.56(0.4 to 0.78)	183.67(95.71 to 320.65)	1.92%(1.75 to 2.08)
Togo	12.11(9.08 to 15.59)	0.33(0.25 to 0.43)	47.21(33.44 to 63.94)	0.56(0.4 to 0.76)	289.78(156.41 to 473.24)	1.97%(1.88 to 2.07)
Tokelau	0.01(0.01 to 0.01)	0.54(0.38 to 0.77)	0.01(0 to 0.01)	0.51(0.36 to 0.72)	-18.7(-39.79 to 7.41)	-0.14%(-0.5 to 0.23)
Tonga	0.29(0.21 to 0.4)	0.29(0.21 to 0.4)	0.39(0.28 to 0.54)	0.37(0.26 to 0.5)	36.59(-0.32 to 90.21)	0.59%(0.33 to 0.86)
Trinidad and Tobago	11.83(11.03 to 12.64)	0.98(0.92 to 1.05)	22.43(17.07 to 28.77)	1.61(1.23 to 2.07)	89.56(41.98 to 146.7)	1.57%(1.43 to 1.7)
Tunisia	133.04(102.45 to 169.4)	1.59(1.23 to 2.03)	267.79(188.95 to 376.13)	2.26(1.6 to 3.18)	101.28(26.04 to 206.71)	0.99%(0.92 to 1.05)
Turkey	1256.3(907.32 to 1693.43)	2.19(1.58 to 2.95)	1649.48(1285.88 to 2109.45)	1.97(1.54 to 2.52)	31.3(-12.55 to 90.76)	-0.56%(-0.77 to -0.35)
Turkmenistan	49.12(45.57 to 52.74)	1.33(1.23 to 1.43)	43.86(33.68 to 57.64)	0.85(0.65 to	-10.71(-32.67 to 17.88)	-1.76%(-1.94 to

				1.12)		-1.59)
Tuvalu	0.04(0.03 to 0.06)	0.46(0.35 to 0.59)	0.06(0.04 to 0.07)	0.47(0.36 to 0.6)	32.87(-4.4 to 84.55)	-0.14%(-0.3 to 0.02)
Uganda	143.88(108.31 to 184.43)	0.83(0.63 to 1.07)	262.85(181.95 to 368.87)	0.61(0.42 to 0.85)	82.68(22.39 to 165.87)	-1.73%(-1.99 to -1.46)
Ukraine	2452.9(2213.98 to 2715.14)	4.65(4.2 to 5.15)	1333.47(871.52 to 1879.05)	3.1(2.02 to 4.36)	-45.64(-64.56 to -22.03)	-1.99%(-2.22 to -1.76)
United Arab Emirates	7.65(4.73 to 11)	0.41(0.25 to 0.59)	34.58(24.47 to 46.73)	0.36(0.25 to 0.49)	352.24(199.66 to 655.65)	-1%(-1.42 to -0.58)
United Kingdom	1047.42(1015.81 to 1070.61)	1.83(1.77 to 1.87)	937.78(877.78 to 976.36)	1.38(1.29 to 1.44)	-10.47(-14.36 to -7.4)	-1.06%(-1.23 to -0.89)
United Republic of Tanzania	207.19(147.88 to 286.51)	0.8(0.57 to 1.11)	321.3(222.93 to 489.72)	0.55(0.38 to 0.84)	55.08(8.68 to 117.29)	-1.55%(-1.68 to -1.43)
United States of America	4198.67(4024.92 to 4304.45)	1.65(1.58 to 1.69)	4620.32(4339.97 to 4835.98)	1.39(1.3 to 1.45)	10.04(6.26 to 13.9)	-0.83%(-0.95 to -0.72)
United States Virgin Islands	1.42(1.14 to 1.83)	1.34(1.07 to 1.73)	1.84(1.3 to 2.5)	2.15(1.52 to 2.91)	29.78(-16.72 to 86.37)	1.55%(1.34 to 1.76)
Uruguay	186.84(173.33 to 202.45)	5.95(5.52 to 6.45)	131.25(118.35 to 146.72)	3.85(3.48 to 4.31)	-29.75(-37.65 to -21.07)	-1.45%(-1.6 to -1.3)
Uzbekistan	249.66(219.11 to 284.18)	1.19(1.05 to 1.36)	174.73(138.92 to 219.78)	0.51(0.41 to 0.64)	-30.01(-44.65 to -11.74)	-2.46%(-3.01 to -1.91)
Vanuatu	0.33(0.21 to 0.46)	0.21(0.14 to 0.3)	0.76(0.53 to 1.05)	0.24(0.17 to 0.34)	132.5(69.56 to 214.58)	0.16%(0.06 to 0.25)
Venezuela (Bolivarian Republic of)	251.75(237.94 to 264.36)	1.34(1.27 to 1.41)	567.95(431.86 to 732.96)	2.13(1.62 to 2.75)	125.6(70.51 to 192.85)	0.7%(0.41 to 0.98)
Viet Nam	514.33(405.04 to 669.44)	0.75(0.59 to 0.98)	1354.66(994.42 to 1811.08)	1.35(0.99 to 1.81)	163.38(81.17 to 279.16)	2%(1.93 to 2.08)
Yemen	131.49(81.93 to 186.79)	0.96(0.6 to 1.37)	325(200.19 to 473.3)	0.97(0.6 to 1.41)	147.17(64.97 to 288.41)	-0.09%(-0.22 to 0.03)
Zambia	60.55(45.22 to 76.52)	0.76(0.57 to 0.96)	151.21(74.83 to 345.17)	0.77(0.38 to 1.77)	149.72(27.98 to 445.2)	-0.27%(-0.48 to -0.07)
Zimbabwe	84.49(64.68 to 105.59)	0.82(0.63 to 1.02)	164.59(124.52 to 221.38)	1.06(0.8 to 1.42)	94.8(43.07 to 169.8)	0.81%(0.56 to 1.06)

Data in parentheses are 95% uncertainty intervals for cases and rates, and 95% confidence intervals for EAPC.
Rate is expressed as per 100,000 population.
